# Do antithrombotic drugs have a role in migraine prevention? A systematic review

**DOI:** 10.1111/head.14917

**Published:** 2025-02-24

**Authors:** Federico De Santis, Matteo Foschi, Michele Romoli, Vincenzo Mastrangelo, Chiara Rosignoli, Agnese Onofri, Simona Sacco, Raffaele Ornello

**Affiliations:** ^1^ Department of Biotechnological and Applied Clinical Sciences University of L'Aquila L'Aquila Italy; ^2^ Department of Neuroscience Maurizio Bufalini Hospital, AUSL Romagna Cesena Italy; ^3^ Neurology Unit “Infermi” Hospital, AUSL Romagna Rimini Italy

**Keywords:** antithrombotic, aura, microembolism, migraine, preventive treatment, prothrombotic state

## Abstract

**Objectives:**

To explore the available evidence on the role of antithrombotics as migraine preventive medication.

**Background:**

In clinical practice, the use of antithrombotic drugs in individuals with migraine is sometimes considered, especially in the case of frequent auras, association with patent foramen ovale, or prothrombotic states. This paper systematically reviews evidence on antithrombotic agents’ efficacy for migraine prevention.

**Methods:**

We performed a systematic literature search on PubMed and Scopus including observational and interventional studies focused on antiplatelets or anticoagulants as preventive treatments for migraine. The search included studies published until June 30th, 2024. Ongoing trials on Clinicaltrials.org were also explored. Quality assessment used the Cochrane Risk of Bias 2 (RoB‐2) tool for randomized controlled trials (RCTs) and the Risk Of Bias In Non‐randomized Studies of Interventions (ROBINS‐I) for observational studies. The protocol was registered in the International Prospective Register of Systematic Reviews (PROSPERO identifier CRD42024501531).

**Results:**

Out of 1854 records, we found 12 RCTs and 8 observational studies investigating the impact of antithrombotic drugs in migraine prevention. Due to heterogeneity of data, a meta‐analysis was not feasible. RCTs tested acetylsalicylic acid (ASA) alone (seven), ASA in combination with other preventive treatments (two), clopidogrel (one), dual antiplatelet treatment (one), and vitamin K antagonists (one). Observational studies tested ASA (three), vitamin K antagonists (three), and clopidogrel (two). No clear evidence of efficacy was found for the overall population of individuals with migraine. Limited evidence from old RCTs—not specifically addressing the role of antithrombotic drugs for migraine prevention—and observational studies showed a potential improvement of migraine with the use of antiplatelet agents, mostly ASA, in special populations, including males, individuals with migraine with aura, and those with patent foramen ovale.

**Conclusions:**

Evidence supporting the effectiveness of antithrombotic drugs as a preventive treatment for patients with migraine is insufficient. As preliminary data show potential improvements in special populations in whom those agents act indirectly by ameliorating vascular function, RCTs are worth conducting.

## Plain Language Summary

Research suggests that some of the mechanisms of migraine might include alterations in blood clotting, leading researchers to question whether antithrombotic (i.e., blood‐thinning) medications might help with preventing migraine attacks. In this study, we looked at all the research on the use of blood‐thinning drugs to prevent migraine attacks to see if these drugs work for people with migraine in general, as well as for people with specific types of migraine (e.g., those with aura, those with certain heart or neurological conditions). Our results suggest that for most people with migraine, blood‐thinning drugs probably do not help with preventing migraine attacks; however, these drugs might help some people with specific conditions or types of migraine, such as those with aura or concomitant blood clotting disorders.

**Abbreviations**
ASA: acetylsalicylic acid
MRI: magnetic resonance imaging
PFO: patent foramen ovale
RCT: randomized controlled trial
SD: standard deviation
VKA: vitamin K antagonists
WMH: white matter hyperintensities

## INTRODUCTION

Migraine is a common neurological disorder, representing the second cause of disability worldwide and the first cause in women aged <50 years.^1^, ^2^ Migraine is acknowledged as a risk factor for vascular diseases,^3^ as its presence increases the risk of ischemic stroke and cardiac ischemic disease up to two‐fold, especially for migraine with aura.^4^, ^5^ The mechanisms linking migraine to cerebrovascular events are unclear but probably rely on a peculiar vulnerability of cerebral tissue to ischemia^6^ that may be precipitated by other conditions, such as comorbidity with prothrombotic factors, hypercoagulability, and microembolism.^7^ Antiplatelet agents that have an anti‐inflammatory effect, like acetylsalicylic acid (ASA), are effective for the acute management of migraine attacks.^8^, ^9^ Antiplatelet agents have also been tested in migraine prevention. Their action may be mediated by their anti‐inflammatory properties, as well as their effect in inhibiting platelet aggregation and microemboli formation that may play a role in migraine aura.^7^

Additionally, the concern for increased cardiovascular risk may lead clinicians to consider antithrombotics in some circumstances, such as in the presence of a patent foramen ovale (PFO), thrombophilic alterations, or white matter hyperintensities (WMH) at brain magnetic resonance imaging (MRI).^10^

The primary objective of the present systematic review was to summarize the available evidence on the efficacy and safety of antithrombotic agents for migraine prevention. The secondary objective of the systematic review was to assess the efficacy and safety of antithrombotic agents for migraine prevention in specific subgroups, including patients with migraine with aura, PFO, or WMHs at brain MRI.

The ultimate goal of the systematic review was to delineate a patient profile that may respond to such treatments or whether it is worth further investigation.

## METHODS

### Search strategy and selection criteria

We conducted a systematic review following the Preferred Reporting Items for Systematic Reviews and Meta‐Analyses (PRISMA) guidelines^11^, ^12^ and the Cochrane Handbook for Systematic Reviews of Interventions.^13^ The protocol was registered in the International Prospective Register of Systematic Reviews (PROSPERO identifier CRD42024501531).

We performed a literature search on articles published in English up to June 30th, 2024, on PubMed and Scopus, using the following search string: migraine AND (prevent* OR prophyla*) AND (aspirin OR acetylsalicyl* OR ticlopidine OR clopidogrel OR antiplatelet* OR anticoagul* OR antithromb* OR warfarin OR dabigatran OR rivaroxaban OR apixaban OR edoxaban OR acenocoumarol OR phenprocoumon OR heparin). Cilostazol and dipyridamole were excluded from the search due to their ability to generate migraine‐like attacks.^14^, ^15^ We also excluded other nonsteroidal anti‐inflammatory drugs not approved as antithrombotic agents, including naproxen, ibuprofen, ketoprofen, and indomethacin.

We selected articles that fulfilled the following inclusion criteria:

- Design: observational (prospective or retrospective) or interventional studies (including randomized controlled trials [RCTs]).
- Population: patients with a diagnosis of any type of migraine.
- Intervention: antiplatelets or anticoagulants used as preventive agents (including ASA, clopidogrel, ticlopidine, dabigatran, rivaroxaban, apixaban, edoxaban, warfarin, acenocoumarol, phenprocoumon, and heparin).
- Comparator (only for interventional studies): placebo or other preventive treatment.
- Outcome: migraine frequency (attacks or number of headache/migraine days), consumption of symptomatic drugs, and migraine‐related disability as reported by patients’ headache diaries.

We excluded articles not fulfilling inclusion criteria with the following labels:

- Wrong type of publication: editorial, comments, letters, reviews.
- Wrong population: not including patients with migraine.
- Wrong intervention: drugs not used as antithrombotics.
- Wrong outcome: outcomes different from migraine frequency, consumption of acute treatments, or migraine‐related disability.
- Not pertinent: the article was too generic and did not contain data of interest for the review (fulfilling multiple exclusion criteria).

As a first step, four authors (R.O., F.D.S., M.F., M.R.) independently screened all the records for title and abstracts using Rayyan Systematic Reviews web‐based tool.^16^ Then, the same authors selected the articles after examining full texts. We also conducted a manual search of references of the retrieved full texts. We did not perform a search of gray literature as we meant to focus on peer‐reviewed data. Disagreements on eligibility were resolved by consensus among all the involved authors.

Additionally, we searched for ongoing trials on Clinicaltrials.org investigating the preventive action of antithrombotic drugs on patients with migraine, using the same keywords as the main literature search.

### Data extraction

Data extraction was conducted by two authors independently (M.F. and M.R.) using an electronic spreadsheet with the following pre‐specified variables for the RCTs: first author, year, population, types of intervention, numbers of patients in experimental and control groups, outcomes, and adverse events. For the observational studies, we extracted the following variables: first author, year, design, sex proportions, mean age (age range), proportion of patients with aura, mean headache/migraine frequency, type of intervention, comparator, follow‐up duration, outcome, and main results.

The RCTs were classified according to the drugs tested, while observational studies were classified according to both the tested drugs and the populations—overall population of individuals with migraine, individuals with migraine with aura, and individuals with migraine and PFO.

### Statistical analysis

We planned meta‐analyses of data comparing the same drug with the same outcomes across RCTs or observational studies.

### Quality assessment

The quality of RCTs was assessed with the Cochrane Risk of Bias 2 (RoB‐2) tool,^17^ while the quality of observational studies was assessed with the Risk Of Bias In Non‐randomized Studies of Interventions (ROBINS‐I) tool.^18^

## RESULTS

**FIGURE 1:**
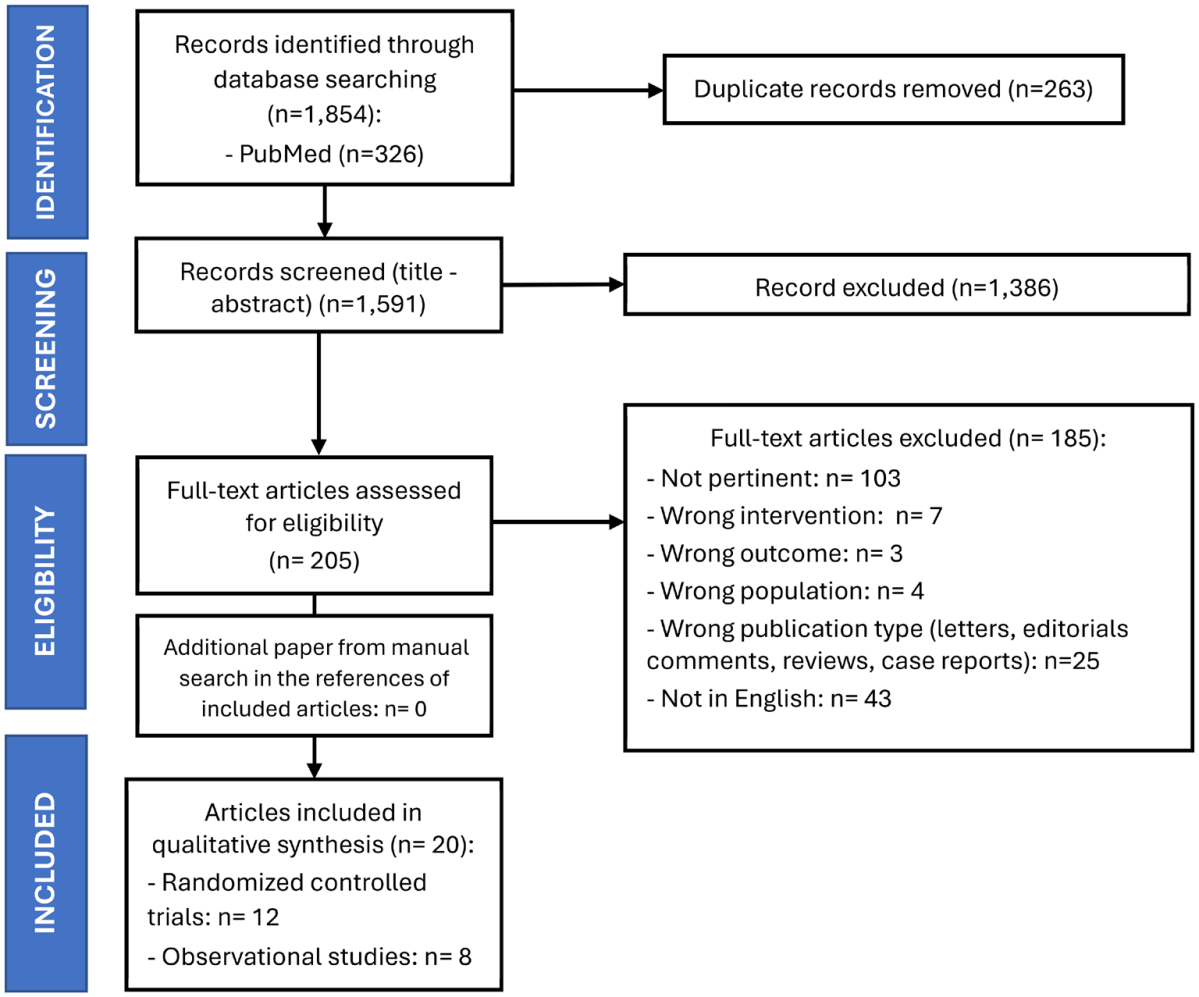
The Preferred Reporting Items for Systematic Reviews and Meta‐Analyses (PRISMA) 2020 flowchart of study selection. [Colour figure can be viewed at wileyonlinelibrary.com]

**FIGURE 2:**
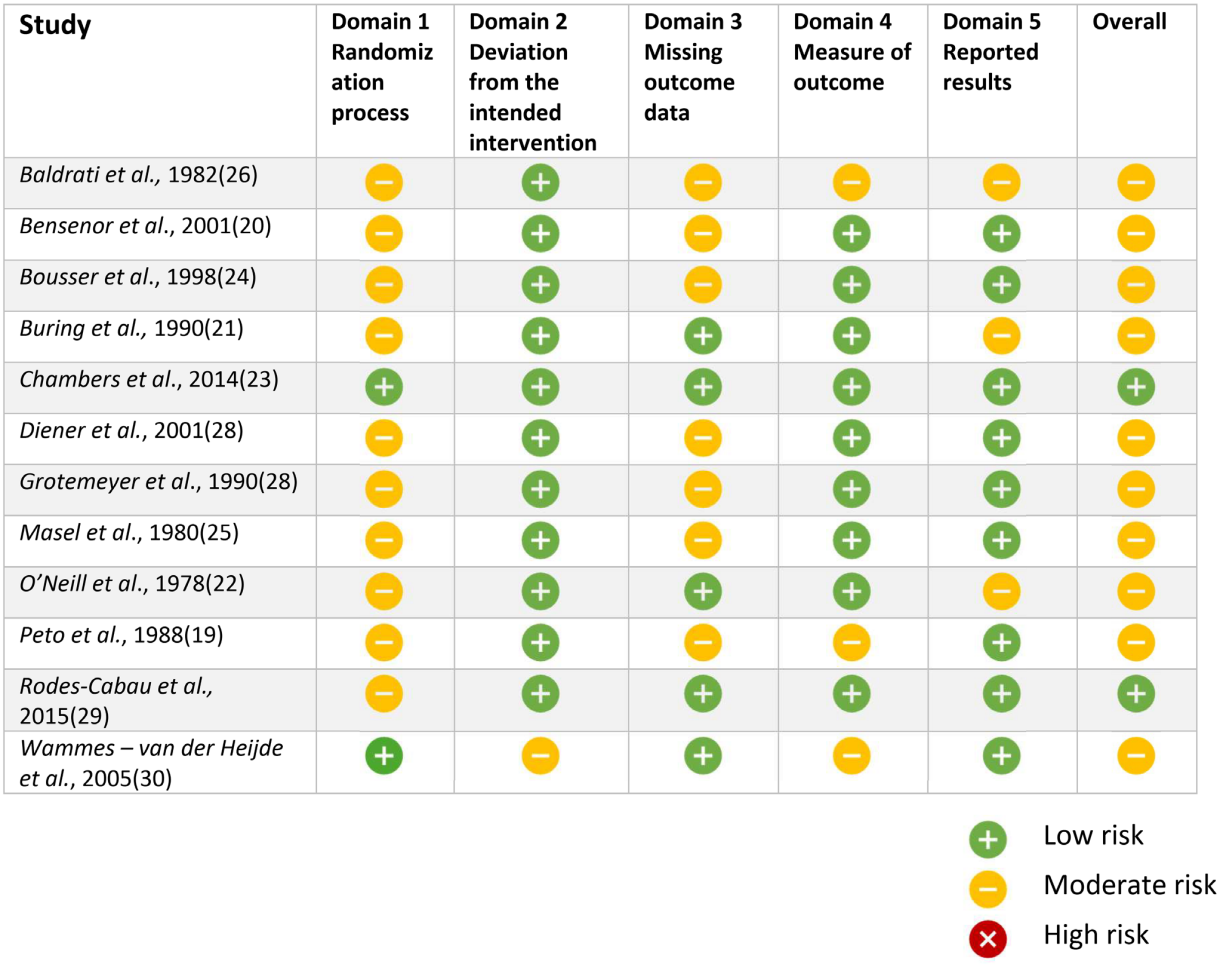
Risk of bias analysis of randomized controlled trials included in the present systematic review. The analysis was performed according to the Cochrane Risk of Bias 2 (RoB‐2) tool. [Colour figure can be viewed at wileyonlinelibrary.com]

**FIGURE 3:**
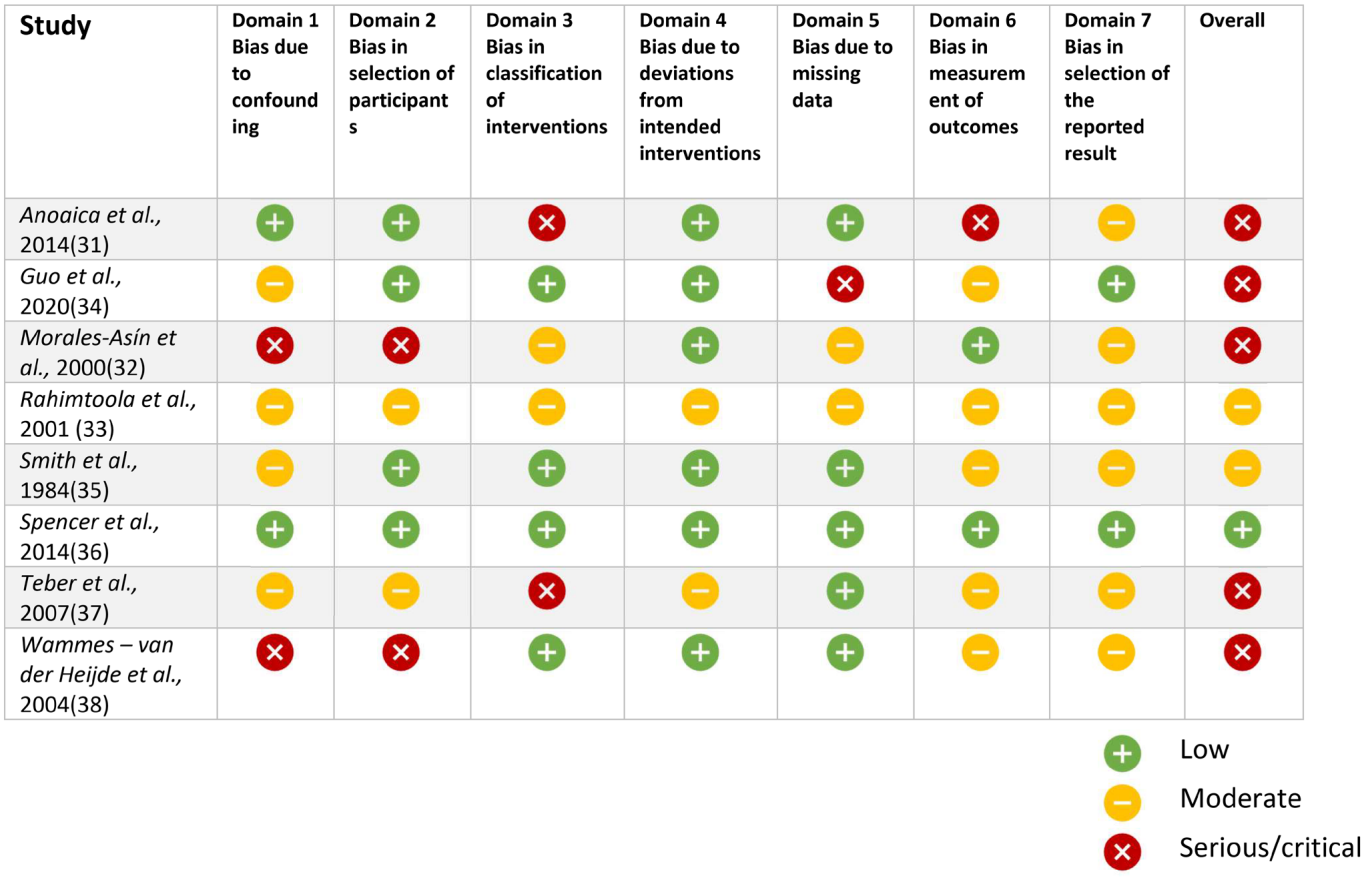
Risk of bias analysis of observational studies included in the present systematic review. The analysis was performed according to the Cochrane Risk Of Bias In Non‐randomized Studies of Interventions (ROBINS‐I) tool [Colour figure can be viewed at wileyonlinelibrary.com].

The initial search found 1854 records. After removal of duplicates, 1591 articles were screened for titles and abstracts; 205 records were deemed relevant for full‐text evaluation (Figure 1 and Table S1). After full‐text assessment, 20 records fulfilled our inclusion criteria, including 12 RCTs^19^, ^20^, ^21^, ^22^, ^23^, ^24^, ^25^, ^26^, ^27^, ^28^, ^29^, ^30^ and 8 observational studies (five prospective and three retrospective)^31^, ^32^, ^33^, ^34^, ^35^, ^36^, ^37^, ^38^ (Tables 1, 2, 3). Two of the 12 RCTs (16.6%) were judged at low risk of bias, while 10 (83.3%) showed some concerns for bias, mostly in the methods of the randomization process and for missing data. Among the eight observational studies, one (12.5%) was considered at low risk of bias, while five (62.5%) were considered at serious and two (25.0%) at moderate risk of bias: the main reasons are issues in the correction of confounding factors, heterogenous and not standardized classification of the interventions, and measurement of outcomes (Figures 2 and 3, see Tables S2 and S3 for detailed information on the bias risk judgment).

**TABLE 1:** Main results of antithrombotic drugs for migraine prevention in randomized controlled trials in the overall population of patients with migraine.

First author, year	*N* (%) females	Age, years, mean (SD)	Frequency of migraine at baseline	Randomization/design	Duration of intervention	Experimental group	Control group	Outcomes	Results (active vs. placebo)	Adverse events
ASA versus no treatment										
Peto et al., 1988^19^	0 (healthy male physicians)	N.R.	N.R.	Open label, parallel groups	6 years	ASA 500–300 mg daily (*N* = 3429)	No treatment (avoid aspirin) (*N* = 1710)	Number of patients reporting migraine attacks for which medical attention was sought	Less patients with migraine attacks in ASA group 197/3429 (5.7%) vs. no treatment 276/1710 (16.1%), *p* < 0.001	670 (19.5%) stopped ASA in the first year: 494 for medical reasons/adverse events (gastrointestinal symptoms first cause)
ASA alone versus placebo										
Benseñor et al., 2001^20^	1001 (100)	51.5 (5.40) ASA vs. 51.3 (4) placebo	≥1 monthly migraine attack	Double‐blind, parallel groups	12–36 months	ASA 100 mg once daily (*N* = 525)	Placebo (*N* = 476)	Number of patients reporting improvement in questionnaires: 1) Reduction in headache frequency at 12 and 36 months 2) Reduction in headache severity at 12 and 36 months 3) Reduction in headache duration at 12 and 36 months 4) Reduction in headache‐related incapacitation at 12 and 36 months	Reduction trend in all the outcomes without statistical significance. 1) 12 months: 189 (53.5%) vs. 172 (50.3%); 36 months: 271 (59.6%) vs. 235 (56.4%) 2) 12 months: 177 (50.4%) vs. 154 (44.9%) 36 months: 240 (52.5%) vs. 212 (50.5%) 3) 12 months: 156 (44.4%) vs. 136 (40.0%); 36 months: 216 (47.7%) vs. 186 (44.8%) 4) 12 months: 191 (54.9%) vs. 169 (49.9%); 36 months: 254 (56.8%) vs. 223 (53.7%)	N.R.
Buring et al., 1990^21^	0 (only male healthy physicians)	53.2 (9.5)	Unknown (migraine history not required)	Double‐blind, parallel groups	Up to 60 months	ASA 325 mg once daily (*N* = 11,037)	Placebo (*N* = 11,034)	Any report of migraine attacks during follow‐up	661 (6.0%) patients on ASA vs. 818 (7.4%; *p* < 0.001) on placebo reported migraine at some time after randomization	N.R.
O'Neill et al., 1978^22^	5 (41.6%)	Age range 18–53	>1 day/month	Double‐blind, crossover trial	3 months each during two consecutive periods	ASA 650 mg twice daily (*N* = 12)	Placebo (*N* = 12)	Rate of responders (>50% reduction in frequency over 3 months)	75% (9/12 cases, 3 with classic migraine and 6 with common migraine) responders in ASA (*p* < 0.001)	3 patients with gastritis
CLP monotherapy versus placebo										
Chambers et al., 2014^23^	60 (75.0 %)	45 (12)	4–15 headache days/month	Triple‐blind, parallel groups	3 months	CLP 75 mg once daily (*N* = 35)	Placebo (*N* = 36)	1) 3‐month change in number of headache days 2) 3‐month MIDAS and HIT‐6 score	1) Headache days fell by 1.9 days on CLP and by 1.8 days on placebo (not significant) 2) Mean (SD) MIDAS score 30.2 (22.7) CLP vs. 30.2 (27.8) placebo. Mean (SD) HIT‐6 score 54.7 (4.6) CLP vs. 55.0 (5.2)	3 with CLP, 3 with placebo (including bruises and nosebleed)
ASA combined with other drugs versus placebo										
Bousser et al., 1998^24^	26 (68.0 %)	39.6 (13.6)	3–15 days/month	Double‐blind, crossover	8 weeks	ASA 80mg + DHE 10mg daily (*N* = 38)	Placebo (*N* = 38)	1) Number of attacks reduction after 8 weeks 2) Number of patients with 20% reduction in frequency	1) 31% fewer attacks with DHE + ASA (11.5 ± 6.2) than with placebo (16.6 ± 9.9), *p* = 0.003 2) 22 patients in DHE + ASA vs. 7 placebo (*p* < 0.02)	Adverse events in 6 DHE + ASA vs. 1 in placebo
Masel et al., 1980^25^	23 (92.0%)	Range 21–64	>1/month	Double‐blind, crossover	3 months each during two consecutive periods	ASA 325 mg twice daily + DIPY 25 mg three times a day (*N* = 25)	Placebo (*N* = 25)	Change in: 1) frequency (headaches for month), 2) intensity (0–100 pain scale) and 3) activity level (0–5 activity scale) In the total population and in the subpopulation of HAP and not HAP subgroups.	Significant improvement in median value of: 1) frequency (total 1.33 active vs. 2.33 placebo; HAP 0.67 vs. 4.00, *p* < 0.01) 2) intensity (total 50.00 vs. 77.00, *p* < 0.01; HAP 2.00 vs. 3.00 *p* < 0.02) vs. pretreatment. 3) activity (total 3.00 vs. 2.00, *p* < 0.05; HAP 3.00 vs. 2.00, *p* < 0.05). (17/25) 68% subjective improving on active medication	N.R.

Abbreviations: ASA, acetylsalicylic acid; CLP, clopidogrel; DHE, dihydroergotamine; DIPY, dipyridamole; HAP, hyper‐aggregable; HIT‐6, six‐item Headache Impact Test; MIDAS, Migraine Disability Assessment; N.R., not reported, SD, standard deviation; vs. versus.

ASA was the most investigated drug, followed by vitamin K antagonists (VKAs, e.g., acenocoumarol) and clopidogrel. Other antithrombotic drugs that were investigated included dipyridamole in association with ASA.

Due to the high heterogeneity among available studies in the selection criteria for included patients, outcome definitions, and duration of follow‐up, meta‐analyses were not feasible.

We found RCTs and observational studies performed in the overall population of individuals with migraine, in individuals with migraine with aura, and in those with migraine and PFO. We did not find any completed or ongoing study on the effect of antithrombotic drugs on individuals with migraine and thrombophilic alterations or in those with migraine and WMHs at brain MRI.

### Antithrombotic drugs for migraine prevention in the overall population of individuals with migraine

#### Randomized controlled trials

##### Antithrombotic drugs versus no treatment

One RCT^19^ (Table 1) explored the effect of ASA 300–500 mg daily, compared with no treatment, on migraine in 5139 healthy male physicians; participants treated with ASA had a lower proportion of migraine attacks (5.7% vs. 16.1%, *p* < 0.001) over 6 years of follow‐up compared with those not treated with ASA.

##### Antithrombotic drugs versus placebo

Overall, we found six RCTs^20^, ^21^, ^22^, ^23^, ^24^, ^25^ investigating antithrombotic drugs alone (four) or in combination with another non‐antithrombotic agent (two) against placebo in migraine prevention (Table 1). Of the four RCTs on antithrombotic drugs alone, three tested ASA^20^, ^21^, ^22^ and one tested clopidogrel,^23^ while the two RCTs^24^, ^25^ on combined treatments tested the combinations of ASA with dihydroergotamine and with dipyridamole, respectively. One RCT,^20^ including 1001 females with migraine, showed that daily ASA 100 mg was not superior to placebo in decreasing frequency, duration, severity, and disability of the attacks at 12 and 36 months. A large RCT^21^ including 22,071 healthy male physicians found that those treated with ASA 325 mg daily reported a lower proportion of migraine attack occurrence compared with those treated with placebo (6.0% vs. 7.4%; relative risk 0.80, 95% confidence interval 0.72–0.88; *p* < 0.001) over up to 5 years of follow‐up. A crossover RCT^22^ included 12 patients (seven males and five females) with ≥1 monthly attack of migraine treated with ASA 650 mg twice daily for 3 months and placebo for 3 months; nine of the 12 patients had a 50% reduction in monthly migraine attacks during treatment with ASA compared with the placebo period.

One RCT^23^ investigated the effect of a 3‐month treatment with clopidogrel 75 mg daily compared with placebo in 71 patients (75.0% female) with episodic migraine. This study did not reveal any significant benefit from clopidogrel compared with placebo for reduction of headache days or for improvement in migraine disability (measured by the Migraine Disability Assessment questionnaire score) and headache impact (measured by the six‐item Headache Impact Test score).

Referring to RCTs combining two drugs (Table 1) with a crossover design, one RCT^24^ examined ASA 80 mg in association with dihydroergotamine 10 mg against placebo in 38 participants with episodic migraine (3–15 days/month). Participants treated with ASA and dihydroergotamine reported a lower number of attacks at 8 weeks of follow‐up compared with those treated with placebo, at a mean (standard deviation [SD]) of 11.5 (6.2) versus 16.6 (9.9) (*p* = 0.003) without significant difference in duration, severity, or mean consumption of acute drug treatment. The other RCT^25^ compared ASA 325 mg twice daily plus dipyridamole 25 mg thrice daily with placebo in 25 participants; a significant improvement was found in migraine frequency in the active medication arm as compared to placebo (median frequency 1.3 vs. 2.3 monthly attacks; *p* < 0.01; median intensity 50.0 vs. 77.0 on a 0–100 scale; *p* < 0.01).

##### Antithrombotic drugs versus other preventive agents

We found three RCTs^26^, ^27^, ^28^ and an open‐label trial^30^ comparing an antithrombotic agent with an active treatment for migraine prevention (Table 2). In the three RCTs, the antithrombotic drug was ASA and the comparator was a β‐blocker (metoprolol in two studies, propranolol in one study). As detailed in Table 2, the overall evidence favored the β‐blocker over ASA (up to 500 mg thrice a day) in the two studies^27^, ^28^ involving metoprolol 200 mg on the primary outcome, expressed as a rate of 50% responders. The study^26^ involving propranolol 1.8 mg/kg daily as an active comparator was neutral on the migraine index reduction (a custom index calculated from severity, frequency, and duration of the attacks). In an open‐label head‐to‐head comparison^30^ including 12 females, acenocoumarol (target international normalized ratio 1.5–2.0) failed to show a significant preventive efficacy on 12‐week change in monthly migraine attacks over propranolol (80–160 mg daily).

**TABLE 2:** Main results of head‐to‐head randomized controlled trials of antithrombotic drugs versus other migraine preventive drugs for migraine prevention in the overall population of patients with migraine.

First author, year	*N* (%) females	Age, years, mean (SD)	Frequency of migraine at baseline	Randomization/design	Duration of intervention	Experimental group	Control group	Outcomes	Results (active vs. placebo)	Adverse event
Baldrati et al., 1982^26^	10 (83.3%)	Age range 18‐49	At least 2 attacks/month	Double‐blind, crossover	3 months each during 2 consecutive periods	ASA (13.5 mg/kg) daily (*N* = 6)	PRP (1.8 mg/kg) daily (*N* = 6)	Mean reduction of MI (severity × frequency × duration of the latest 3 months)	Effectiveness of both ASA and PRP were comparable: MI mean reduction of 65.2% with ASA vs. 64.8% with PRP from the pretreatment period. (no significant difference)	Adverse event in 6 patients in each group (palpitation, gastric distress, constipation)
Diener et al., 2001^27^	219 (81.1%)	Females: 38.9 Males: 41.8	2–6 attacks/month	Double‐blind, parallel groups	16 weeks	ASA 300 mg daily (*N* = 135)	MTP 200 mg daily (*N* = 135)	Rate of responders (>50% reduction in migraine frequency)	29.6% ASA vs. 45.2% MTP of responders (significant, without *p* specified)	Adverse events 37 ASA (mainly gastrointestinal) vs. 73 MTP (skin, psychiatric, cardiac)
Grotemeyer et al., 1990^28^	23 (82.1%)	31.1 (10)	4–8 attacks/month	Double‐blind, crossover	12 weeks	ASA 500 mg three times a day (*N* = 21)	MTP 200 mg once (*N* = 21)	Rate of responders (>50% reduction in frequency compared to run‐in period)	Number of responders: 3 (14.0%) in ASA vs. 14 (67.0%) in MTP (*p* < 0.01) Mean attacks frequency reduction vs. baseline in ASA (26% ± 22%) vs. MTP (50% ± 18%)	Adverse event dropout 5 in ASA (gastrointestinal) vs. 2 in MTP (drowsiness)
Wammes van der Heijde et al., 2005^30^	12 (100%)	41.5	3–8 attacks/month	Randomized open controlled crossover study	26 weeks (12 + 2 wash out + 12)	Acenocoumarol (INR 1.5‐2) (*N* = 5)	PRP 80 mg once (up to two times) a day (*N* = 7)	Change in monthly migraine attacks from baseline during the treatment period with acenocoumarol vs. PRP	50% decrease in migraine monthly attacks in 2 patients treated with acenocoumarol vs. 5 treated with PRP (not significant).	

Abbreviations: ASA, acetylsalicylic acid; INR, international normalized ratio; MTP, metoprolol, MI, Migraine Index; PRP, propranolol; SD, standard deviation; vs., versus.

#### Observational studies

Three observational studies evaluated the effect of VKAs on migraine prevention with heterogeneous methods and non‐univocal results (Table 3). One retrospective study^32^ including 166 participants with headache (66 migraine and 100 non‐migraine) showed that acenocoumarol—prescribed for indications different from headache—led to an improvement, defined as ≥60% reduction in headache frequency, of all types of headache, being more pronounced in migraine compared to non‐migraine headache (63.0% vs. 38.0%, respectively; *p* = 0.004). Another retrospective study,^33^ extracting data from a prescription database, included 92 individuals who started VKAs or low‐dose ASA for clinical conditions different from headache; individuals with migraine were identified as those having received a previous prescription of migraine acute symptomatic drug (ergotamine or sumatriptan). The study showed a higher reduction in the consumption of symptomatic drugs for migraine in individuals treated with VKAs compared with those treated with ASA (−40% vs. −4.7%, *p* = 0.04). Finally, a prospective case series^38^ investigated the potential preventive effect of acenocoumarol on migraine in four individuals with thromboembolic risk factors. Again, acenocoumarol was prescribed for indications unrelated to migraine. The study observed that anticoagulation led to migraine improvement in only two patients, both of whom had factor V Leiden heterozygosity. The other two participants, who had elevated factor VIII levels and increased von Willebrand factor activity, did not experience any migraine‐related benefits from anticoagulation.

**TABLE 3:** Main results of observational studies of antithrombotic drugs for migraine prevention in the overall population of patients with migraine.

First author, year	Design	*N*	Aura, *n* (%)	Mean headache/migraine frequency (range)	Intervention	Comparator	Follow‐up duration	Outcome	Main results
General population of patients with migraine									
Morales‐Asín et al., 2000^32^	Retrospective	166:66 (39.7%) with migraine and 100 (60.3%) with non‐migraine type headache	N.R.	N.R.	Acenocoumarol for non‐neurological indication	None	Mean (SD, range) duration of oral anticoagulation: 4.56 (0.28, 0.1–23) years	Headache improvement (reduction in the frequency of headache by at least 60%)	Improvement in 42/66 (63%) patients with migraine and 38/100 (38%) patients with non‐migraine type headache (*p* = 0.004) Vomiting and migraine severity were significantly more frequent in patients who improved than in those who did not improve.
Rahimtoola et al., 2001^33^	Retrospective	92 (32 treated with oral VKA and 60 with low‐dose ASA) patients with prescription of more than one abortive migraine drug and concomitant prescription of ASA or VKA	N.A.	N.A.	Acenocoumarol/phenprocoumon	Low‐dose ASA	Mean (range) duration of oral anticoagulation 13.7 (4.7–23.2) months, mean (95% CI) duration of low‐dose ASA 30.3 (23.5–37.0) months	Percentage reduction in the therapeutic intensity (defined daily doses per patient per month of ergotamine and sumatriptan use)	Ergotamine and sumatriptan use decreased from 6.4 doses/month prior to anticoagulant treatment to 3.0 doses/month during oral anticoagulation treatment, compared with a reduction from 5.2 doses/month to 4.4 for the low‐dose ASA cohort (*p* = 0.05). The therapeutic intensity of ergotamine and sumatriptan use was significantly decreased by 40% for the oral anticoagulation cohort, compared with 4.7% for the low‐dose ASA acid cohort (*p* = 0.04)
Smith et al., 1984^35^	Prospective	50 headache patients (≥4 attacks/ month) including 40 with migraine	N.R.	At least 4 attacks/month	ASA 250 mg daily for 2 months	ASA for 2 days	2 months	Reduction in frequency and severity after 4 and 8 weeks. Decrease in platelet aggregability.	In ASA: 20 (40.0%) responders (11 mild, 4 strong improvement, 5 headache free) 28 (56.0%) no response 2 (4.0%) worsening of attacks Decrease in aggregability in 31 (62%), without a significant improvement in headache frequency and intensity
Teber et al., 2007^37^	Prospective	25 children with migraine	N.A.	>3 attacks/month	ASA 100 mg daily	PRP 1–2 mg/kg/day	6 months	Decrease in number of attacks per month; headache severity; and headache duration	PRP compared to ASA reduced significantly the frequency (percentage of patients with decreased attack, ASA mean [SD] 63% [19%] vs. propranolol 83% [23%], *p* = 0.043); non‐significant reduction in duration and severity
Wammes van der Heijde et al., 2004^38^	Prospective	4 patients with migraine, thromboembolic risk of factors and previous reported benefit on migraine frequency during previous use of anticoagulant	N.A.	N.A.	Low‐dose acenocoumarol for 12 weeks, INR 1.5–2	None	22 weeks	Difference in attack frequency between the treatment period and the run‐in period	84% and 73% reduction of attack in 2 patients (both with factor V Leiden heterozygosity); 2 patients discontinued treatment, no effect All 4 patients had one or more thromboembolic risk factor

Abbreviations: ASA, acetylsalicylic acid; CI, confidence interval; INR, international normalized ratio; N.A., not available; N.R., not reported; PRP, propranolol; SD, standard deviation; vs., versus.

Two prospective studies evaluated the effect of ASA on individuals with migraine. The first one^37^ included 25 children and adolescents with migraine (age range 6–16 years) and showed a lower reduction in monthly migraine attacks in participants treated with ASA compared with those treated with propranolol (mean [SD] −63% [19%] vs. –83% [23%], *p* = 0.043) after 6 months of follow‐up. The second one^35^ did not show any headache improvement after 2 months of treatment with ASA 250 mg against placebo on an adult population of 50 individuals (46 with migraine and four with “vascular headache”).

### Antithrombotic drugs for migraine prevention in individuals with migraine with aura

We found one observational retrospective study^31^ (Table 4) comparing the efficacy and tolerability of ASA (100–300 mg daily) with other prophylactic therapies used for migraine in 203 participants with migraine with aura. In this study, 88.4% of patients treated with ASA reported a reduction in frequency and duration of aura, compared to 59.3% of those treated with other prophylactic drugs (*p* < 0.001).

**TABLE 4:** Main results from studies of antithrombotic drugs for migraine prevention in the specific subgroups of patients with migraine.

First author, year	Design	*N*	Aura, *n* (%)	Headache/migraine frequency, mean (SD)	Intervention	Comparator	Follow‐up length	Outcome	Main results
Migraine with aura									
Anoaica et al., 2014^31^	Retrospective	203 patients with migraine with aura: 95 (46.8%) treated with ASA, 108 (53.2%) treated with other prophylactic drugs	203/203 (100)	Mean pre‐treatment migraine frequency: 3.83 (1.57)/week Mean pre‐treatment aura duration: 36.21 (19.80) min	ASA started from 300 mg/day for a minimum of 4 months, then reduced to 200 and 100 mg/day	Other prophylactic migraine therapy	4 months (minimum) to 194 months	Reduction in migraine frequency	88/95 patients (88.4%) treated with ASA referred positive results vs. 64/108 (59.3%) who underwent other prophylactic treatments (*p* < 0.001). Significant reduction in migraine frequency (from a mean [SD] of 3.83 [1.57] pre‐treatment to 1.38 [0.87] after treatment; *p* < 0.001) and in aura duration (from a mean [SD] of 36.21 [19.80] pre‐treatment to 22.0 [15.5] after‐treatment; *p* < 0.001) 7/95 patients (7.3%) had no migraine
Migraine and PFO									
Guo et al., 2020^34^	Prospective	30 patients with drug‐refractory migraine and PFO	9 (34.6)	Baseline headache frequency: 6.17 (3.93)/month Baseline attack duration: 13.62 (13.98)/h	CLP 75 mg daily added to existing prophylactic regimen for 3–6 months	None	3–6 months	Reduction vs. baseline of: 1) frequency, 2) attack duration, 3) Visual analog score 4) MIDAS; maintained for 6 months in 12 patients	Significant reduction in: 1) Headache migraine frequency: 3 months follow‐up: 3.28 (2.67)/month, 6 months follow‐up: 2.75 (1.35)/month 2) Headache attack duration: 3 months follow‐up: 7.36 (7.33)/h. 6 months follow‐up: 6.95 (5.36)/h 3) 6.17 (3.93) vs. 3.28 (2.67)/month, *p* = 0.003 4) 13.62 (13.98) vs. 7.36 (7.33)/h, *p* = 0.049
Spencer et al., 2014^36^	Prospective	15 patients with migraine (all with PFO and r‐l shunt)	12/15 (80)	2/week – daily	CLP 75 mg added to existing prophylactic migraine regimen for 4 weeks	None	4 weeks	Reduction (%) in the frequency of migraine symptoms	4/15 had >50% reduction migraine. 8/15 had no migraine. 1/15 had no change in migraine symptoms
Rodes‐Cabau et al., 2015^29^	RCT	171 patients receiving PFO closure	N.R.	No history of migraine	DAPT (ASA 80 mg + CLP 75 mg) For 3 months after PFO closure	SAPT (ASA 80 mg + placebo)	3 months	1) Monthly new onset migraine attacks within the 3 months following PFO closure for clinical indication 2) incidence of new attacks 3) severity of new onset attacks	1) Mean number 0.4 DAPT vs. 1.4 placebo, difference: −1.02 days (95% CI −1.94 to−0.10 days); incident risk ratio 0.61, 95% CI 0.41 to 0.91; *p* = 0.04 2) 27(15.8%) patients: 8 (9.5%) CLP group vs. 19(21.8%) placebo group, difference −12.3% (*p*=0.03) 3) zero disabling attacks in DAPT vs. 7 (36.8%) in placebo (*p* = 0.046)

Abbreviations: ASA, acetylsalicylic acid; CI, confidence interval; CLP, clopidogrel; DAPT, dual antiplatelet treatment; MIDAS, Migraine Disability Assessment; N.R., not reported; PFO, patent foramen ovale; RCT, randomized controlled trial; SAPT, single antiplatelet treatment; SD, standard deviation; vs., versus.

### Antithrombotic drugs for migraine prevention in individuals with migraine and PFO

Two observational prospective studies^34^, ^36^ and one RCT^29^ investigated the role of antithrombotic drugs in patients with concomitant migraine and PFO (Table 4). The first study^34^ included 30 participants with refractory migraine (defined in this case as poor responders [≥2 attacks/month] to two or more classes of preventive treatments), and presence of PFO, treated with clopidogrel 75 mg once daily added to existing preventive therapy and followed up from 3 to 6 months without a comparator. The study found, in the patients with 3 months follow‐up (*n* = 26), a significant reduction of headache frequency (from a mean [SD] of 6.17 [3.93] to 3.2 [2.6] attacks/month, *p* = 0.003) and of headache attack duration (from a mean [SD] of 13.6 [13.9] to 7.3 [7.3] h, *p* = 0.049) together with visual analog score and migraine disability assessment scores (from a mean [SD] of 6.3 [1.9] to 4.7 [1.2], *p* < 0.001; from a mean [SD] of 22.1 [7.1] to 16.0 [5.9], *p* = 0.001, respectively).

The second study^36^ was a 4‐week open‐label observational study on a sample of 15 patients with migraine, PFO, and confirmed right‐to left shunt, without a history of stroke. The study showed that 13/15 patients (86.6%) reported a ≥50% decrease in migraine monthly frequency on clopidogrel treatment compared with baseline.

Lastly, one RCT investigated dual antiplatelet treatment with ASA plus clopidogrel compared with ASA alone in people (*n* = 171) without a history of migraine, who were undergoing PFO closure for indications different from migraine.^29^ Results showed that ASA plus clopidogrel, compared to ASA plus placebo was associated with a protective effect on new migraine onset after PFO closure (9.5% vs. 21.8%; *p* = 0.03).

### Ongoing trials

We found three ongoing RCTs investigating antithrombotic agents in patients with migraine registered on ClinicalTrials.gov [Table 5]. All studies are investigating populations of patients with migraine and PFO. Two studies are investigating the efficacy of antiplatelets (ASA and/or clopidogrel) versus PFO closure in reducing migraine frequency (50% responder rate and migraine cessation at 12‐month follow‐up, respectively). The third RCT will compare three arms of treatment with different antithrombotic agents (ASA 100 mg, clopidogrel 75 mg and rivaroxaban 20 mg) to an active comparator (metoprolol 25 mg twice a day) in individuals with migraine and PFO, having as primary outcome the 50% responder rate at 3 months.

**TABLE 5:** Ongoing randomized controlled trials investigating the efficacy of antithrombotic drugs for migraine prevention.

Trial, NCT	Status	Year of expected completion (Country)	Enrollment (*N* estimated)	Design	Population (inclusion criteria)	Intervention(s)	Comparator(s)	Outcome (primary)
Comparison of the Effect of Medication Therapy in Alleviating Migraine With Patent Foramen Ovale (COMPETE) NCT05546320 https://clinicaltrials.gov/study/NCT05546320?cond=Migraine&intr=aspirin&rank=10	Recruiting	2022–2025 (China)	1000	Parallel groups	Migraine with diagnosis >1 year PFO presence Age 18–65 years	A) ASA 100 mg qd vs. B) CLP 75 mg qd vs. C)Rivaroxaban 20 mg qd	Metoprolol 25 mg twice a day	Efficacy: 3 months responder rate as 50% reduction in MMD Safety: AE
COMParison of the EffecT of dEvice Closure in Alleviating Migraine With PFO (COMPETE‐2) NCT05561660 https://clinicaltrials.gov/study/NCT05561660?cond=Migraine&intr=Acetylsalicylic%20acid&rank=8	Recruiting	2022–2025 (China)	460	Parallel groups	Migraine with diagnosis >1 year PFO presence Age 18–65 years	PFO closure	ASA 200 mg/qd for 6 months	Efficacy: 12 months responder rate as 50% reduction in MMD Safety: AE
Effectivity and Safety of PFO Closure vs. Medicine in Alleviating Migraine (SPRING) NCT04946734 https://clinicaltrials.gov/study/NCT04946734?cond=Migraine&intr=aspirin&rank=9	Recruiting	2021–2025 (China)	440	Parallel groups	Migraine PFO presence with right to left shunt Age 16–60 years	PFO closure + ASA 100 mg 6 months after closure + CLP 75 mg for 1 month after closure Triptans for the acute phase	ASA and CLP as preventive Triptans as acute treatment	Efficacy: complete migraine cessation in 12 months

Abbreviations: AE, adverse event; ASA, acetylsalicylic acid; CLP, clopidogrel; MMD: monthly migraine days; NCT, National Clinical Trial; PFO, patent foramen ovale; qd, once a day (from the Latin *quaque die*); vs. versus.

## DISCUSSION

Our systematic review found insufficient evidence to support the efficacy of antithrombotic medications for migraine prevention in the overall migraine population. Regarding subgroups of individuals with migraine, there are no data to support their use in special populations such as individuals with migraine with aura, individuals with migraine and PFO, and individuals with migraine and WMHs on brain MRI. Nevertheless, it is worth mentioning that available studies do not provide robust evidence to support the ineffectiveness of antithrombotic agents in the overall population of migraine and in special populations of individuals with migraine. The available studies were heterogeneous with respect to the drugs that were tested, their dosages, and the outcomes that were considered, so that they could not be pooled in meta‐analyses. Besides, most studies had an unclear risk of bias (Figure 2). Due to the heterogeneity and risk of bias, even positive results from those studies should be interpreted with caution and should not form the evidence basis for prescription in clinical practice. While in the overall population of individuals with migraine, considering also availability of new specific agents with high efficacy and excellent safety profile, it is not worth testing the efficacy of antithrombotics currently, there is a rationale to further investigate them in special populations. In fact, vascular mechanisms such as microembolism or prothrombotic activities have been implicated in some special situations in individuals with migraine. Microembolism may lead to aura occurrence in PFO as well as prothrombotic factors.^7^, ^39^ Thus, in special populations, it is worth further investigating the potential benefit of antithrombotic agents.

The available RCTs had methodological limitations, including heterogeneous outcomes that did not reflect current standards for migraine prevention trials, suboptimal study procedures, and lack of sample size calculations in most cases. The largest RCT^21^ did not pre‐specify migraine prevention among the aims of its comparison between ASA and placebo.

Antithrombotic drugs decreased migraine frequency compared to placebo only in studies not primarily designed to assess their effect on migraine prevention.^19^, ^21^ These studies included only male participants, tested higher dosages than those used for cardiovascular prevention, and sometimes reported a considerable prevalence of adverse events.^19^ Conversely, a large study on an exclusively female cohort failed to demonstrate significant efficacy of ASA over placebo for migraine prevention.^20^ Head‐to‐head comparisons between ASA and alternative migraine preventive agents (β‐blockers) indicated that antithrombotic medications are not suitable as preventive treatment.^26^, ^27^, ^28^, ^30^ Two RCTs involving ASA combinations with dihydroergotamine or dipyridamole showed efficacy against placebo.^24^, ^25^ However, dihydroergotamine might be poorly tolerated,^40^ while the combination of ASA with dipyridamole might increase the risk of hemorrhagic complications.

Despite the overall ineffectiveness of antithrombotic drugs for migraine prevention, it could be valuable to assess their potential efficacy in selected populations, including those with migraine with aura, PFO, WMHs, or thrombotic and vascular risk factors comorbid with migraine. To date, no RCTs have specifically targeted these patient groups. Pre‐specified subgroup analyses of new RCTs or large observational studies might reveal interactions between migraine and vascular comorbidities for which antithrombotic drugs could provide benefit.

Together with RCTs, the observational studies included in our analysis also exhibited low methodological quality, relying on small patient cohorts and demonstrating a moderate‐to‐high risk of bias. Some suggested potential benefit from antithrombotic drugs in individuals with migraine with aura or those with PFO,^34^, ^36^ possibly due to prevention of microembolism and platelet activation mediated by shear stress.^31^ However, observational evidence lacks control for the placebo effect, which is substantial in migraine.^41^

Ongoing RCTs are exploring various antithrombotic regimens for migraine prevention in participants with both migraine and PFO. This population may be of particular interest, given the lack of clear evidence on PFO closure for migraine prevention in the general migraine population. RCTs and a related meta‐analysis have shown some benefit of PFO closure only in individuals with migraine with aura.^42^ However, PFO closure can cause new‐onset migraine in up to 15% of individuals^43^, ^44^ and carries risks associated with surgical intervention. The risk–benefit profile of VKA for migraine prevention remains unclear due to the retrospective design and small sample sizes of available studies.

Previous systematic reviews investigated the role of antiplatelet drugs for migraine prevention.^45^, ^46^ The first^45^ concluded for a potential effect on migraine frequency of ASA in high doses (>325 mg daily) mainly adopted for cardiovascular prevention. The second review^46^ focused on P2Y12 inhibitors (including clopidogrel and ticagrelor) in migraine prevention, highlighting a potential role of those drugs against migraine onset after PFO closure. Nevertheless, these reviews failed to identify an optimal dosage or a specific subgroup of patients most likely to benefit from the treatment. Notably, both reviews underscored the heterogeneity of available RCTs, precluding unequivocal conclusions.

Despite inconclusive evidence from RCTs, some guidelines^47^, ^48^, ^49^, ^50^, ^51^ cautiously suggest ASA^21^, ^28^ at a dosage of 100–300 mg daily or even anticoagulants for migraine prevention, albeit with a low class of evidence. These recommendations are based upon expert consensuses, given the limitations and heterogeneity in existing evidence.

Considering the tolerability of antithrombotic medications is crucial, given their potential for long‐term adverse events such as intracranial and extracranial bleeding and peptic ulcers.^52^ The concerns surrounding tolerability, coupled with modest efficacy, raise questions about the suitability of antithrombotic agents as preventive drugs for migraine.

Evidence supports heightened platelet aggregability in patients with migraine compared to those without migraine, as well as a higher cardiovascular risk. It is plausible that the action of antithrombotic agents is directed toward specific platelet and endothelium alterations pathways, including shear‐stress aggregation and related release of vasoactive agents^53^, ^54^, ^55^—including serotonin, nitric oxide, endothelin, and prostaglandins—that might be more prevalent in individuals with migraine than in the general population. Evidence supports heightened platelet aggregability in patients with migraine compared to those without migraine as well as a higher cardiovascular risk.^7^, ^56^ Additionally, migraine—and especially migraine with aura—might be associated with coagulation disorders,^7^, ^57^, ^58^, ^59^ even if some prevalence studies have not confirmed this association.^60^, ^61^ Hypercoagulability leading to clot formation in small vessels, or alterations in coagulation resulting in the formation of microemboli, may contribute to migraine‐associated phenomena, including cortical spreading depolarization.^7^, ^62^ Potential target populations for antithrombotic therapy in migraine prevention include patients with a high vascular risk profile and concomitant migraine, as well as those with migraine exhibiting specific characteristics related to vascular pathogenesis (such as aura and PFO), a minority of the population with migraine and potentially difficult to recruit into dedicated RCTs.

The strengths of our systematic review lie in its comprehensive approach, encompassing all antithrombotic drugs tested for migraine prevention. A notable limitation was our inability to pool data from studies into a meta‐analysis due to heterogeneity in outcome measurements and included populations. While a patient‐level meta‐analysis could have addressed intriguing research questions, we opted against it due to the age of many RCTs and observational studies, potentially posing challenges in data retrieval from the original authors.

## CONCLUSIONS

**FIGURE 4:**
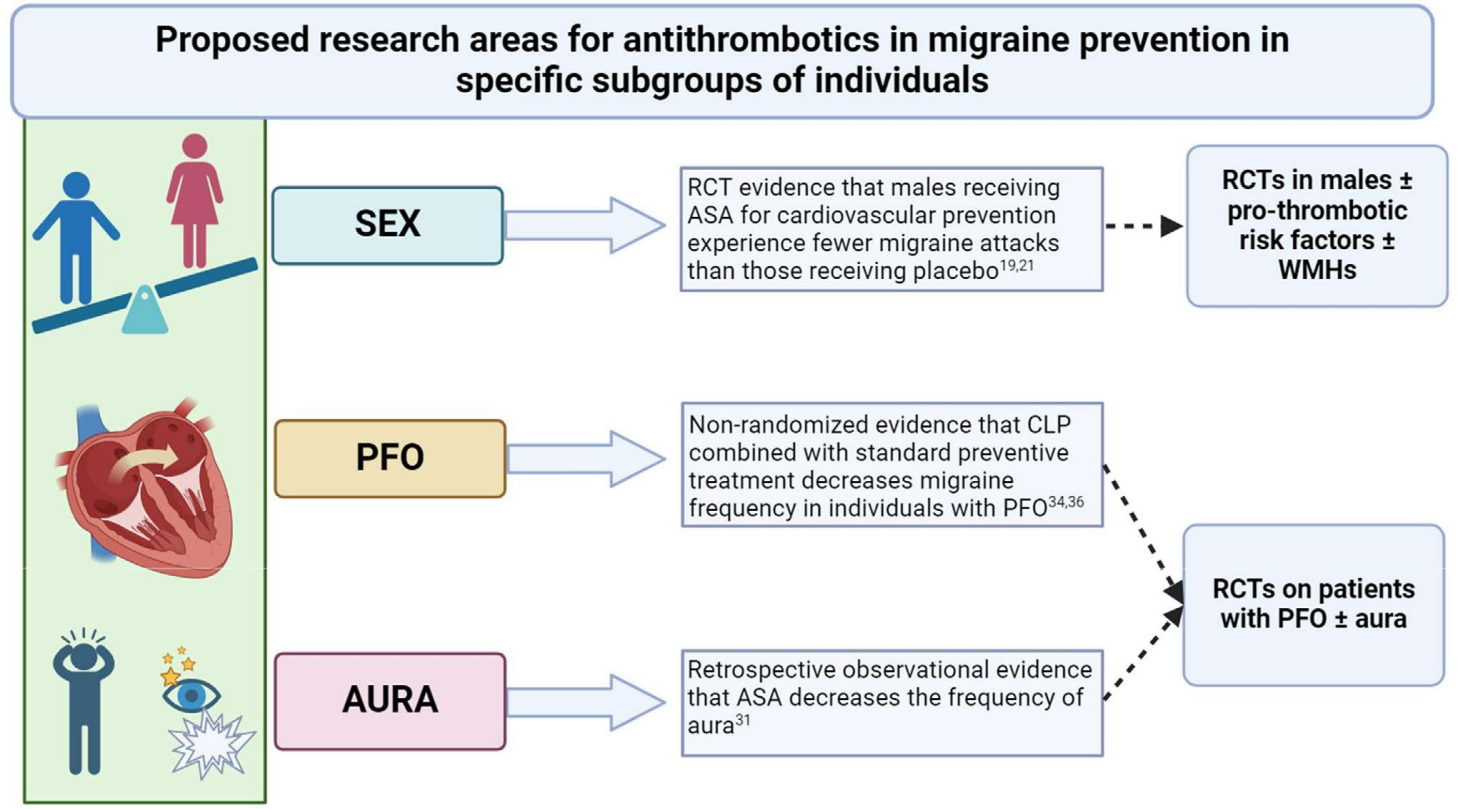
Promising areas for further research on antithrombotic applications in migraine. ASA, acetylsalicylic acid; CLP, clopidogrel; PFO, patent foramen ovale; RCT, randomized controlled trial; WMH, white matter hyperintensities (Created with BioRender.com). [Colour figure can be viewed at wileyonlinelibrary.com]

This systematic review highlighted the inadequacy of evidence supporting the effectiveness of antithrombotic agents as a preventive treatment for individuals with migraine. Despite this overall conclusion, there might be a rationale to further study in rigorous RCTs of the use of antiplatelet agents in individuals with migraine with aura and/or those with PFO or hypercoagulability (Figure 4).

## AUTHOR CONTRIBUTIONS

**Federico De Santis:** Conceptualization; data curation; formal analysis; investigation; methodology; validation; visualization; writing – original draft; writing – review and editing. **Matteo Foschi:** Conceptualization; data curation; formal analysis; methodology; validation; visualization. **Michele Romoli:** Conceptualization; data curation; formal analysis; investigation; methodology; supervision; validation; visualization. **Vincenzo Mastrangelo:** Data curation; formal analysis; investigation; methodology; validation; visualization. **Chiara Rosignoli:** Investigation; methodology; validation; visualization. **Agnese Onofri:** Investigation; methodology; validation; visualization. **Simona Sacco:** Conceptualization; investigation; methodology; project administration; supervision; validation; visualization; writing – review and editing. **Raffaele Ornello:** Conceptualization; data curation; formal analysis; investigation; methodology; project administration; supervision; validation; writing – original draft; writing – review and editing.

## FUNDING INFORMATION

No fundings to declare for this work.

## CONFLICT OF INTEREST STATEMENT

**Simona Sacco** declares personal fees as speaker or advisor from Abbott, Allergan‐Abbvie, AstraZeneca, Boheringer, Eli Lilly, Lundbeck, Novartis, NovoNordisk, Pfizer, and Teva; she reports having received research grants from Novartis and Uriach; she is president of the European Stroke Organization, Editor‐in‐chief of *Cephalalgia* and *Cephalalgia Reports*, and Assistant Editor for *Stroke*. **Raffaele Ornello** reports personal fees as speaker or advisor from AbbVie, Eli Lilly, Novartis, Pfizer and Teva, and non‐financial support from AbbVie, Eli Lilly, Novartis, and Teva; he is Associate Editor in “Headache and Neurogenic Pain” for *Frontiers in Neurology* and Junior Editorial Board Member for *The Journal of Headache and Pain*. **Federico De Santis, Matteo Foschi, Michele Romoli, Vincenzo Mastrangelo, Chiara Rosignoli**, and **Agnese Onofri** declare no conflicting interests.

## Supporting information


Table S1.

Table S2.
