# Supplementary material for: Do antithrombotic drugs have a role in migraine prevention? A systematic review
Source: Headache. 2025 Feb 24;65(4):709–27. doi: 10.1111/head.14917 (PMC11951400; doi:10.1111/head.14917)
Supplement: Supplementary file 1 — Table S1. [file HEAD-65-709-s001.docx]

| N | AUTHORS | YEAR | TITLE | DOI | FULL-TEXT INCLUDED (YES=1; NO=0) | REASON for exclusion |
| --- | --- | --- | --- | --- | --- | --- |
| 1 | Ruscheweyh; R. et al. | 2022 | The headache registry of the German Migraine and Headache Society (DMKG): baseline data of the first 1;351 patients | 10.1186/s10194-022-01447-3 | 0 | Not pertinent |
| 2 | Ezzati; A. et al. | 2022 | Predictive models for determining treatment response to nonprescription acute medications in migraine: Results from the American Migraine Prevalence and Prevention Study | 10.1111/head.14312 | 0 | Not pertinent |
| 3 | Lee; S. et al. | 2023 | Trends in healthcare utilisation of patients with migraine in South Korea: A retrospective observational study using Health Insurance Review and Assessment Service National Patient Sample data from 2010 to 2018 | 10.1136/bmjopen-2021-059926 | 0 | Not pertinent |
| 4 | Santos; P.S.F. et al. | 2022 | Consensus of the Brazilian Headache Society (SBCe) for prophylactic treatment of episodic migraine: part II | 10.1055/s-0042-1755320 | 0 | Not pertinent |
| 5 | Luo; G.-G. | 2022 | Update advance in migraine and patent foramen ovale | 10.3969/j.issn.1672-6731.2022.02.006 | 0 | Not in English |
| 6 | Kristoffersen; E.S. et al. | 2021 | The management and clinical knowledge of headache disorders among general practitioners in Norway: a questionnaire survey | 10.1186/s10194-021-01350-3 | 0 | Not pertinent |
| 7 | **Wang; F. et al.** | **2022** | **Platelet P2Y12 Inhibitor in the Treatment and Prevention of Migraine: A Systematic Review and Meta-Analysis** | **10.1155/2022/2118740** | **1** |  |
| 8 | Eller; M. et al. | 2022 | Migraine management: an update for the 2020s | 10.1111/imj.15843 | 0 | Not pertinent |
| 9 | Kalkman; D.N. et al. | 2023 | Migraine and cardiovascular disease: What cardiologists should know | 10.1093/eurheartj/ehad363 | 0 | Not pertinent |
| 10 | Biglione; B. et al | 2020 | Aspirin in the Treatment and Prevention of Migraine Headaches: Possible Additional Clinical Options for Primary Healthcare Providers | 10.1016/j.amjmed.2019.10.023 | 0 | Wrong publication type |
| 11 | Guo; Y. | 2021 | Response to "comment on Ã¢ â‚¬ Clopidogrel can be an effective complementary prophylactic for drug-refractory migraine with patent foramen ovale'" | 10.1136/jim-2021-001852 | 0 | Wrong publication type |
| 12 | Haghdoost; F. et al. | 2021 | Comment on 'Clopidogrel can be an effective complementary prophylactic for drug-refractory migraine with patent foramen ovale' | 10.1136/jim-2020-001687 | 0 | Wrong publication type |
| 13 | MacHado-Duque et al, | 2021 | Prescription patterns of antimigraine drugs | 10.46997/REVECUATNEUROL30100050 | 0 | Not pertinent |
| 14 | Pristipino; C. et al, | 2021 | Long-term benefits and risks in patients after persistent foramen ovale closure: A contemporary approach to guide clinical decision making | 10.33963/KP.15817 | 0 | Wrong intervention/drug |
| 15 | Scuteri; D. et al. | 2021 | Progress in the treatment of migraine attacks: From traditional approaches to eptinezumab | 10.3390/ph14090924 | 0 | Wrong intervention/drug |
| 16 | Yu; S. et al. | 2020 | Migraine treatment and healthcare costs: Retrospective analysis of the China Health Insurance Research Association (CHIRA) database | 10.1186/s10194-020-01117-2 | 0 | Wrong intervention/drug |
| 17 | Ailani; J. et al | 2021 | The American Headache Society Consensus Statement: Update on integrating new migraine treatments into clinical practice | 10.1111/head.14153 | 0 | Not pertinent |
| 18 | Jenkins; B. | 2020 | Migraine management | 10.18773/austprescr.2020.047 | 0 | Not pertinent |
| 19 | Kouremenos; et al. | 2019 | Consensus of the Hellenic Headache Society on the diagnosis and treatment of migraine | 10.1186/s10194-019-1060-6 | 0 | Not pertinent |
| 20 | Ashina; M. et al. | 2021 | Migraine: integrated approaches to clinical management and emerging treatments | 10.1016/S0140-6736(20)32342-4 | 0 | Not pertinent |
| 21 | **Guo; Y. et al.** | **2020** | **Clopidogrel can be an effective complementary prophylactic for drug-refractory migraine with patent foramen ovale** | **10.1136/jim-2020-001342** | **1** |  |
| 22 | Saldanha; et al. | 2021 | Management of primary headaches during pregnancy; postpartum; and breastfeeding: A systematic review | 10.1111/head.14041 | 0 | Not pertinent |
| 23 | Meier; B. | 2020 | Patent foramen ovale with a license to kill | 10.1007/s00063-019-0561-z | 0 | Not in English |
| 24 | Lucas; C. | 2021 | Migraine with aura | 10.1016/j.neurol.2021.07.010 | 0 | Not pertinent |
| 25 | Kawata; et al. | 2021 | Understanding the migraine treatment landscape prior to the introduction of calcitonin gene-related peptide inhibitors: Results from the Assessment of TolerabiliTy and Effectiveness in MigrAINe Patients using Preventive Treatment (ATTAIN) study | 10.1111/head.14053 | 0 | Not pertinent |
| 26 | Gazerani; P. et al. | 2020 | Sex-Specific Pharmacotherapy for Migraine: A Narrative Review | 10.3389/fnins.2020.00222 | 0 | Not pertinent |
| 27 | Khan; J. et al. | 2021 | Genetics; pathophysiology; diagnosis; treatment; management; and prevention of migraine | 10.1016/j.biopha.2021.111557 | 0 | Not pertinent |
| 28 | Atianzar; K. et al. | 2017 | Update on the management of patent foramen ovale in 2017: Indication for closure and literature review | 10.15420/usc.2017:18:1 | 0 | Not pertinent |
| 29 | Balaji; O. et al. | 2017 | A complete review of migraine | 10.22159/ajpcr.2017.v10i10.19207 | 0 | Not pertinent |
| 30 | Lipton; R.B.et al. | 2018 | Migraine in America Symptoms and Treatment (MAST) Study: Baseline Study Methods; Treatment Patterns; and Gender Differences | 10.1111/head.13407 | 0 | Not pertinent |
| 31 | Collado; F.S. et al. | 2018 | Patent foramen ovale closure for stroke prevention and other disorders | 10.1161/JAHA.117.007146 | 0 | Wrong intervention/drug |
| 32 | Altamura; C. et al. | 2019 | Right-to-Left Shunt and the Clinical Features of Migraine with Aura: Earlier but Not More | 10.1159/000501544 | 0 | Not pertinent |
| 33 | Kaur; A. et al. | 2017 | Trend of use of different antimigraine medications and awareness of migraine; survey based study in Jalandhar | 10.22159/ajpcr.2017.v10s4.21333 | 0 | Not pertinent |
| 34 | Burch; R. | 2019 | Migraine and Tension-Type Headache: Diagnosis and Treatment | 10.1016/j.mcna.2018.10.003 | 0 | Not pertinent |
| 35 | Ansari; H. and Ziad; S. | 2016 | Drugâ€“Drug Interactions in Headache Medicine | 10.1111/head.12864 | 0 | Not pertinent |
| 36 | Giacoppo; D. et al. | 2018 | Long-term effectiveness and safety of transcatheter closure of patent foramen ovale compared with antithrombotic therapy alone: A meta-analysis of six randomised clinical trials and 3;560 patients with reconstructed time-to-event data | 10.4244/EIJ-D-18-00341 | 0 | Wrong outcome |
| 37 | Osipova; V.V. et al. | 2018 | Diagnosis and Treatment of Migraine: Recommendations of Russian Experts | 10.1007/s11055-018-0608-2 | 0 | Not in English |
| 38 | Thomsen; R.W. et al. | 2019 | Patterns of initial migraine treatment in Denmark: A population-based study | 10.1002/pds.4723 | 0 | Not pertinent |
| 39 | Manolis; A.S. | 2017 | Impact of percutaneous closure of interatrial shunts on migraine attacks: Single-operator series and review of the literature | 10.2174/1574887112666170328124939 | 0 | Not pertinent |
| 40 | Vgontzas; A. and Burch; R. | 2018 | Episodic Migraine With and Without Aura: Key Differences and Implications for Pathophysiology; Management; and Assessing Risks | 10.1007/s11916-018-0735-z | 0 | Not pertinent |
| 41 | Silberstein; S.D. | 2017 | Current management: Migraine headache | 10.1017/S1092852917000864 | 0 | Not pertinent |
| 42 | Terrie; Y.C. | 2017 | Headaches: Wise use of OTC analgesics for prevention and management |  | 0 | Not pertinent |
| 43 | Nietlispach; F. and Meier; B. | 2016 | Percutaneous closure of patent foramen ovale: An underutilized prevention? | 10.1093/eurheartj/ehv376 | 0 | Not pertinent |
| 44 | Baena; C.P. et al. | 2017 | The effectiveness of aspirin for migraine prophylaxis: A systematic review | 10.1590/1516-3180.2016.0165050916 | 0 | Wrong publication type |
| 45 | Sader; E. and Rayhill; M. | 2018 | Headache in Pregnancy; the Puerperium; and menopause | 10.1055/s-0038-1673681 | 0 | Not pertinent |
| 46 | Turk; W.E. et al. | 2017 | Aspirin Prophylaxis for Migraine with Aura: An Observational Case Series | 10.1159/000481252 | 0 | Wrong outcome |
| 47 | Schriever; J. et al. | 2014 | Current state of knowledge and developments in the prophylaxis and acute treatment of migraine | 10.1007/s00103-014-2000-x | 0 | Not in English |
| 48 | Weatherall; M.W. | 2015 | The diagnosis and treatment of chronic migraine | 10.1177/2040622315579627 | 0 | Not pertinent |
| 49 | Jamieson; D.G. | 2014 | What the nonneurologist can do to treat headache | 10.1016/j.otc.2013.10.005 | 0 | Not pertinent |
| 50 | Lee; M.J. et al. | 2016 | The migraine-stroke connection | 10.5853/jos.2015.01683 | 0 | Wrong publication type |
| 51 | Éva Csépány et al. | 2015 | Current migraine pharmacotherapy |  | 0 | Wrong publication type |
| 52 | Krome; S. | 2014 | [Migraine: treatment and prevention]. | 10.1055/s-0033-1353968 | 0 | Not in English |
| 53 | Kung; S.-L. et al. | 2015 | Migraine-like visual aura triggered by a large aneurysm in the left extracranial internal carotid artery with successful prevention of recurrence by the new anticoagulant dabigatran: First case report |  | 0 | Not pertinent |
| 54 | **Chambers; J.B. et al.** | **2014** | **Clopidogrel as prophylactic treatment for migraine: A pilot randomised; controlled study** | **10.1177/0333102414531156** | **1** |  |
| 55 | Messali; A. et al. | 2016 | Direct and Indirect Costs of Chronic and Episodic Migraine in the United States: A Web-Based Survey | 10.1111/head.12755 | 0 | Not pertinent |
| 56 | Jena; S.S. et al. | 2015 | Migraine: Pattern of prescription & adverse drug reaction profile in a tertiary care teaching hospital |  | 0 | Not pertinent |
| 57 | Maggioni; F. et al. | 2016 | Warfarin prophylaxis in migraine without aura but not in primary exercise headache | 10.1007/s13760-015-0527-8 | 0 | Wrong publication type |
| 58 | Lionetto; L. et al. | 2016 | Choosing the safest acute therapy during chronic migraine prophylactic treatment: Pharmacokinetic and pharmacodynamic considerations | 10.1517/17425255.2016.1154042 | 0 | Not pertinent |
| 59 | Harriott; A.M. and Barrett; K.M. | 2015 | Dissecting the Association Between Migraine and Stroke | 10.1007/s11910-015-0530-8 | 0 | Not pertinent |
| 60 | **Rodes-Cabau; J. et al.** | **2015** | **Effect of clopidogrel and aspirin vs aspirin alone on migraine headaches after transcatheter atrial septal defect closure: The CANOA randomized clinical trial** | **10.1001/jama.2015.13919** | **1** |  |
| 61 | Davanzo; R. et al. | 2014 | Breastfeeding and migraine drugs | 10.1007/s00228-014-1748-0 | 0 | Not pertinent |
| 62 | Roceanu; A. et al. | 2014 | Current therapies in episodic migraine management |  | 0 | Not pertinent |
| 63 | **Spencer; B.T. et al.** | **2014** | **A retrospective review of clopidogrel as primary therapy for migraineurs with right to left shunt lesions** | **10.1177/0333102414523845** | **1** |  |
| 64 | Pringsheim; T. et al. | 2012 | Migraine prophylactic guideline summary for primary care physicians - Section IV |  | 0 | Not pertinent |
| 65 | Maggioni; F. et al. | 2012 | Migraine responsive to warfarin: An update on anticoagulant possible role in migraine prophylaxis | 10.1007/s10072-011-0926-4 | 0 | Wrong publication type |
| 66 | Lieba-Samal; D. et al. | 2012 | Knowledge about and use of pharmacological and non-pharmacological headache therapies | 10.1007/s00508-012-0250-x | 0 | Not pertinent |
| 67 | Rizzoli; P.B. | 2012 | Acute and preventive treatment of migraine | 10.1212/01.CON.0000418641.45522.3b | 0 | Not pertinent |
| 68 | Holland; S. et al. | 2012 | Evidence-based guideline update: NSAIDs and other complementary treatments for episodic migraine prevention in adults Report of the quality standards subcommittee of the american academy of neurology and the American headache society | 10.1212/WNL.0b013e3182535d0c | 0 | Wrong publication type |
| 69 | Benemei; S. et al. | 2012 | Atrial septal defect closure and de novo migraine: Exclusive ticlopidine efficacy | 10.1177/0333102412460778 | 0 | Not pertinent |
| 70 | Bray; N.N. et al. | 2013 | Migraine: Burden of disease; treatment; and prevention | 10.1016/j.osfp.2013.01.004 | 0 | Wrong publication type |
| 71 | Russo; A. et al. | 2013 | An unusual case report on the possible role of Warfarin in migraine prophylaxis | 10.1186/2193-1801-2-48 | 0 | Not pertinent |
| 72 | Silberstein; S.D. et al. | 2012 | Evidence-based guideline update: Pharmacologic treatment for episodic migraine prevention in adults report of the quality standards subcommittee of the American academy of neurology and the american headache society | 10.1212/WNL.0b013e3182535d20 | 0 | Wrong publication type |
| 73 | Goadsby; P.J. and Sprenger; T. | 2010 | Current practice and future directions in the prevention and acute management of migraine | 10.1016/S1474-4422(10)70005-3 | 0 | Not pertinent |
| 74 | Martins; I.P. | 2009 | Migraine |  | 0 | Not in English |
| 75 | Moloney; M.F. and Johnson; C.J. | 2011 | Migraine Headaches: Diagnosis and Management | 10.1111/j.1542-2011.2011.00024.x | 0 | Not pertinent |
| 76 | Kurth; T. and Diener; H.-C. and Buring; J.E. | 2011 | Migraine and cardiovascular disease in women and the role of aspirin: Subgroup analyses in the Women's Health Study | 10.1177/0333102411412628 | 0 | Wrong outcome |
| 77 | Teggi; R. et al. | 2010 | Case reports on two patients with episodic vertigo; fluctuating hearing loss and migraine responding to prophylactic drugs for migraine. MeniÃ¨re's disease or migraine-associated vertigo? | | 0 | Wrong publication type |
| 78 | MarkovÃ¡; J. | 2009 | Migraine |  | 0 | Not in English |
| 79 | Buse; D.C. et al. | 2009 | Assessing and managing all aspects of migraine: Migraine attacks; migraine-related functional impairment; common comorbidities; and quality of life | 10.4065/84.5.422 | 0 | Not pertinent |
| 80 | Rapoport; A.M. | 2008 | Medication overuse headache: Awareness; detection and treatment | 10.2165/0023210-200822120-00003 | 0 | Not pertinent |
| 81 | SchÃ¼rks; M. and Diener; H.-C. | 2009 | Closure of patent foramen ovale in the prevention of migraine: Not enough evidence in favor | 10.1038/ncpneuro0971 | 0 | Not pertinent |
| 82 | SchÃ¼rks; V.M. and Diener; H.-C. | 2009 | Migraine therapy in 2009: an update for the primary physician |  | 0 | Not in English |
| 83 | Bamford; C.C. and Tepper; S.J. | 2009 | Daily pharmacologic prophylaxis of episodic migraine | 10.1053/j.trap.2009.03.001 | 0 | Wrong publication type |
| 84 | Rapoport; A.M. | 2008 | Acute and prophylactic treatments for migraine: Present and future | 10.1007/s10072-008-0901-x | 0 | Not pertinent |
| 85 | Demarin; V. et al. | 2008 | Evidence based guidelines for the treatment of primary headaches |  | 0 | Not in English |
| 86 | Bigal; M.E. et al. | 2008 | Chronic migraine in the population: Burden; diagnosis; and satisfaction with treatment | 10.1212/01.wnl.0000323925.29520.e7 | 0 | Not pertinent |
| 87 | Grazzi; L. et al. | 2007 | Chronic headaches: Pharmacological and non-pharmacological treatment | 10.1007/s10072-007-0766-4 | 0 | Not pertinent |
| 88 | **Teber; S. et al.** | **2007** | **Prothrombotic risk factors in childhood migraine and comparison of acetylsalicyclic acid and propranolol in prophylactic therapy** | **10.1055/s-0035-1557367** | **1** |  |
| 89 | Becker; W.J. et al. | 2007 | Migraine treatment |  | 0 | Not pertinent |
| 90 | Hershey; A.D. et al. | 2007 | Headaches | 10.1097/MOP.0b013e3282f1a07f | 0 | Not pertinent |
| 91 | Farinelli; I. et al. | 2007 | Aspirin and tension-type headache | 10.1007/s10194-006-0357-4 | 0 | Wrong population |
| 92 | Johnson; M.P. et al. | 2007 | A pharmacogenomic evaluation of migraine therapy | 10.1517/14656566.8.12.1821 | 0 | Not pertinent |
| 93 | Arulmozhi; D.K. et al. | 2006 | Migraine: Current therapeutic targets and future avenues | 10.2174/157016106776359853 | 0 | Not pertinent |
| 94 | Kennedy; B.M. | 2006 | Migraine: A general approach to prevention and treatment |  | 0 | Not pertinent |
| 95 | Goadsby; P.J. | 2006 | Migraine: Emerging treatment options for preventive and acute attack therapy | 10.1517/14728214.11.3.419 | 0 | Not pertinent |
| 96 | Sedighi; B. et al. | 2006 | Evaluation of self-medication prevalence; diagnosis and prescription in migraine in Kerman; Iran |  | 0 | Not pertinent |
| 97 | [No author name available] | 2006 | Current evidence-based migraine therapy |  | 0 | Not in English |
| 98 | Jelinski; S.E. et al. | 2006 | Clinical features and pharmacological treatment of migraine patients referred to headache specialists in Canada | 10.1111/j.1468-2982.2005.01077.x | 0 | Wrong intervention/drug |
| 99 | Linde; M. | 2006 | Migraine: A review and future directions for treatment | 10.1111/j.1600-0404.2006.00670.x | 0 | Not pertinent |
| 100 | Loj; J. and Solomon; G.D. | 2006 | Migraine prophylaxis: Who; why; and how | 10.3949/ccjm.73.9.793 | 0 | Wrong publication type |
| 101 | Graves; B.W. | 2006 | Management of Migraine Headaches | 10.1016/j.jmwh.2006.01.002 | 0 | Not pertinent |
| 102 | Lucas; C. et al. | 2005 | FRAMIG 2000: Medical and therapeutic management of migraine in France | 10.1111/j.1468-2982.2004.00851.x | 0 | Not pertinent |
| 103 | Pringsheim; T. and Edmeads; J. | 2005 | Effective treatment of migraine |  | 0 | Not pertinent |
| 104 | ZermeÃ±o; F. | 2004 | Salicilic acid in treatment of headaches |  | 0 | Not in English |
| 105 | Limmroth; V. et al. | 2004 | Faster; higher; further. Current thinking on acute and prophylactic treatment of migraine | 10.1007/s00482-004-0362-y | 0 | Not in English |
| 106 | Wenzel; R.G. et al. | 2003 | Over-the-counter drugs for acute migraine attacks: Literature review and recommendations | 10.1592/phco.23.4.494.32124 | 0 | Not pertinent |
| 107 | Gladstone; J.P. and Dodick; D.W. | 2003 | Current and emerging treatment options for migraine and other primary headache disorders | 10.1586/14737175.3.6.845 | 0 | Not pertinent |
| 108 | Martin; V.T. | 2004 | Menstrual migraine: A review of prophylactic therapies | 10.1007/s11916-004-0057-1 | 0 | Not pertinent |
| 109 | Diener; H.-C. | 2002 | Acute therapy and prevention of migraine |  | 0 | Not in English |
| 110 | Cuvellier; J.-C. et al. | 2004 | Drug treatment of migraine in children: State of the art | 10.1016/j.arcped.2004.01.005 | 0 | Not in English |
| 111 | Goadsby; P.J. | 2003 | Migraine: Diagnosis and management | 10.1046/j.1445-5994.2003.00453.x | 0 | Not pertinent |
| 112 | Alves De Souza; J. et al. | 2004 | Remission of refractory chronic cluster headache after warfarin administration: Case report | 10.1590/s0004-282x2004000600029 | 0 | Wrong publication type |
| 113 | Adelman; J.U. and Adelman; R.D. | 2001 | Current options for the prevention and treatment of migraine | 10.1016/S0149-2918(01)80069-2 | 0 | Not pertinent |
| 114 | Limmroth; V. et al. | 1999 | Acetylsalicylic acid in the treatment of headache | 10.1046/j.1468-2982.1999.019006545.x | 0 | Wrong publication type |
| 115 | Gupta; V.K. | 1999 | Does vasopressin mediate the migraine-remitting influence of warfarin? |  | 0 | Not pertinent |
| 116 | **Bensenor; I.M. et al.** | **2001** | **Low-dose aspirin for migraine prophylaxis in women** | **10.1046/j.0333-1024.2001.00194.x** | **1** |  |
| 117 | Krobot; K.J. et al. | 1999 | Migraine prescription density and recommendations. Results of the PCAOM Study | 10.1046/j.1468-2982.1999.019005511.x | 0 | Not pertinent |
| 118 | Wiedemann; B. | 2001 | Aspirin; ergotamine and triptans in the treatment of migraine |  | 0 | Not in English |
| 119 | Parsekyan; D. | 2000 | Migraine prophylaxis in adult patients | 10.1136/ewjm.173.5.341 | 0 | Wrong publication type |
| 120 | **Rahimtoola; H. et al.** | **2001** | **Reduction in the intensity of abortive migraine drug use during coumarin therapy** | **10.1046/j.1526-4610.2001.01141.x** | **1** |  |
| 121 | **Morales-Asi­n; F. et al.** | **2000** | **Patients with acenocoumarol treatment and migraine** | **10.1046/j.1526-4610.2000.00008.x** | **1** |  |
| 122 | Mueller; L. | 2002 | Menstrual migraine: How "mini-prophylaxis" can work for your patient |  | 0 | Not pertinent |
| 123 | Diener; H.C. | 1995 | Migraine prophylaxis by means of low dose acetylsalicylic acid? |  | 0 | Not in English |
| 124 | Ramadan; N.M. et al. | 1997 | Migraine prophylactic drugs: Proof of efficacy; utilization and cost | 10.1046/j.1468-2982.1997.1702073.x | 0 | Wrong publication type |
| 125 | Kolosova; O.A. | 1998 | Acetylsalicylic acid in therapy of migraine |  | 0 | Not in English |
| 126 | Pfaffenrath; V. et al. | 1998 | Treatment of tension-type headache. Recommendations of the German migraine and headache society | 10.1007/s004820050138 | 0 | Not in English |
| 127 | Pfaffenrath; V. and Goes; A. | 1996 | Medical therapy for menstrual migraine | 10.1007/s004820050035 | 0 | Not in English |
| 128 | Russmann; D. et al. | 1993 | Is low dose acetylsalicylic acid effective in the prophylaxis of migraine? |  | 0 | Not in English |
| 129 | Pfaffenrath; V. and Reiter; M. | 1988 | Drug treatment of migraine |  | 0 | Not in English |
| 130 | Buring; J.E. et al. | 1991 | Low-dose aspirin for migraine prophylaxis |  | 1 |  |
| 131 | Grotemeyer; K.-H. et al. | 1991 | Acetylsalicylic acid versus metroprolol in migraine prophylaxis. A double-blind crossover study |  | 0 | Not pertinent/duplicate |
| 132 | Grieb; G. | 1991 | Migraine prophylaxis with acetylsalicylic acid |  | 0 | Not pertinent |
| 133 | Vapaatalo; H. | 1994 | Tolfenamic Acid and Migraine Aspects on Prostaglandins and Leukotrienes | 10.1111/j.1600-0773.1994.tb02004.x | 0 | Wrong intervention/drug |
| 134 | **Grotemeyer KH et al.** | **1990** | **Acetylsalicylic acid vs. metoprolol in migraine prophylaxis--a double-blind cross-over study** | **10.1111/j.1526-4610.1990.hed3010639.x** | **1** |  |
| 135 | Lemaire; V. | 1991 | Low-dose aspirin for migraine prophylaxis |  | 0 | Not pertinent |
| 136 | [No author name available] | 1991 | Acetylsalicylic acid in the prevention of migraine |  | 0 | Not pertinent |
| 137 | [No author name available] | 1990 | Low-dose aspirin for migraine prophylaxis |  | 0 | Not pertinent |
| 138 | Kudrow; D.B. and Kudrow; L. | 1989 | Successful Aspirin Prophylaxis in a Child with Chronic Paroxysmal Hemicrania | 10.1111/j.1526-4610.1989.hed2905280.x | 0 | Wrong population |
| 139 | Spector; R.H. | 1984 | Migraine | 10.1016/0039-6257(84)90205-4 | 0 | Wrong publication type |
| 140 | **Bousser; M.G. et al.** | **1988** | **Combined Low-Dose Acetylsalicylic Acid and Dihydroergotamine in Migraine Prophylaxis: A Double-Blind; Placebo-Controlled Crossover Study** | **10.1046/j.1468-2982.1988.0803187.x** | **1** |  |
| 141 | **Peto; R. et al.** | **1988** | **Randomised trial of prophylactic daily aspirin in British male doctors** | **10.1136/bmj.296.6618.313** | **1** |  |
| 142 | **O'Neill; B.P. and Mann; J.D.** | **1978** | **ASPIRIN PROPHYLAXIS IN MIGRAINE** | **10.1016/S0140-6736(78)92159-1** | **1** |  |
| 143 | Carrieri; P.B. and Orefice; G. and Sorge; F. | 1988 | A double blind placebo controlled trial of indobufen in the prophylaxis of migraine | 10.1111/j.1600-0404.1988.tb05935.x | 0 | Wrong intervention/drug |
| 144 | Masel; B. et al. | 1978 | Clinical trial of platelet inhibition; using aspirin and dipyridamole in migraine prophylaxis |  | 0 | Not pertinent/duplicate |
| 145 | Moeller; H.C. | 1983 | Long-term prophylaxis of migraine with acetylsalicylic acid |  | 0 | Not in English |
| 146 | Pothmann; R. | 1987 | Childhood migraine prophylaxis with calcium antagonist flunarizine and acetylsalicylic acid. A double blind study |  | 0 | Not in English |
| 147 | Kimura; S. et al. | 1986 | A case of hemiplegia migraine treated with low-dose aspirin |  | 0 | Not in English |
| 148 | Fukuda; Y. and Izumikawa; K. | 1988 | Intravenous Aspirin for Intractable Headache and Facial Pain | 10.1111/j.1365-2524.1988.hed2801047.x | 0 | Not pertinent |
| 149 | Andrea; G.D. et al. | 1982 | Platelet Activity in Migraine | 10.1111/j.1526-4610.1982.hed2205207.x | 0 | Not pertinent |
| 150 | Smith; M. et al. | 1984 | Salicylate prophylaxis in migraine |  | 0 | Wrong publication type |
| 151 | Ryan; R.E. | 1981 | Migraine prophylaxis: A new approach |  | 0 | Not pertinent |
| 152 | Joseph; R. et al. | 1985 | Thromboxane Synthetase Inhibition: Potential Therapy in Migraine | 10.1111/j.1526-4610.1985.hed2504204.x | 0 | Wrong publication type |
| 153 | **Baldrati; A. et al.** | **1983** | **Propranolol and acetylsalicylic acid in migraine prophylaxis: Double blind crossover study** | **10.1111/j.1600-0404.1983.tb04561.x** | **1** |  |
| 154 | Bamji; A. | 1987 | Aspirin as prophylaxis against migraine | 10.1136/bmj.294.6574.772 | 0 | Wrong publication type |
| 155 | Noda; S. et al. | 1985 | Successftul treatment of migraine attacks with intravenous injection of aspirin | 10.1136/jnnp.48.11.1187 | 0 | Not pertinent |
| 156 | **Masel; B.E. et al.** | **1980** | **Platelet Antagonists in Migraine Prophylaxis A Clinical Trial Using Aspirin and Dipyridamole** | **10.1111/j.1526-4610.1980.hed2001013.x** | **1** |  |
| 157 | Liu X and Gong Y | 2020 | The Potential Protective Role of Aspirin Against Migraine in Pregnant Women. | 10.12659/MSM.923959 | 0 | Wrong population |
| 158 | Cameron C et al. | 2015 | Triptans in the Acute Treatment of Migraine: A Systematic Review and Network Meta-Analysis. | 10.1111/head.12601 | 0 | Not pertinent |
| 159 | **Anoaica MB et al.** | **2014** | **Acetylsalicylic Acid in migraine with aura prevention - a retrospective study.** | **10.12865/CHSJ.40.02.08** | **1** |  |
| 160 | Tomita H et al. | 2007 | Efficacy of ticlopidine for preventing migraine after transcatheter closure of atrial septal defect with Amplatzer septal occluder: a case report. | | 0 | Not pertinent |
| 161 | Wilmshurst PT et al. | 2005 | Clopidogrel reduces migraine with aura after transcatheter closure of persistent foramen ovale and atrial septal defects. | 10.1136/hrt.2004.047746 | 0 | Wrong population |
| 162 | **Wammes-van der Heijden EA et al.** | **2005** | **Effect of low-intensity acenocoumarol on frequency and severity of migraine attacks.** | **10.1111/j.1526-4610.2005.05028.x** | **1** |  |
| 163 | **Wammes-van der Heijden EA et al.** | **2004** | **A thromboembolic predisposition and the effect of anticoagulants on migraine.** | **10.1111/j.1526-4610.2004.04090.x** | **1** |  |
| 164 | **Diener HC et al.** | **2001** | **A comparative study of oral acetylsalicyclic acid and metoprolol for the prophylactic treatment of migraine. A randomized; controlled; double-blind; parallel group phase III study.** | **10.1046/j.1468-2982.2001.00168.x** | **1** |  |
| 165 | Nelson-Piercy C and De Swiet M | 1996 | Diagnosis and management of migraine. Low dose aspirin may be used for prophylaxis. | 10.1136/bmj.313.7058.691b | 0 | Wrong publication type |
| 166 | Pradalier A and Vincent D | 1992 | [Migraine and non-steroidal anti-inflammatory agents]. |  | 0 | Not pertinent |
| 167 | **Buring JE and Peto R and Hennekens CH** | **1990** | **Low-dose aspirin for migraine prophylaxis.** |  | **1** |  |
| 168 | Keidel; M. | 2011 | New strategies in migraine therapy | 10.1055/s-0038-1628458 | 0 | Not in English |
| 169 | Silberstein; S.D. | 2008 | Treatment recommendations for migraine | 10.1038/ncpneuro0861 | 0 | Not pertinent |
| 170 | Diener; H.-C. et al. | 2008 | Current diagnosis and treatment of migraine | 10.1007/s00482-007-0619-3 | 0 | Not in English |
| 171 | Evers; S. et al. | 2009 | EFNS guideline on the drug treatment of migraine - Revised report of an EFNS task force | 10.1111/j.1468-1331.2009.02748.x | 0 | Wrong publication type |
| 172 | Von Hoffmann; J. and Reuter; U. | 2009 | Therapy and prophylaxis of migraine |  | 0 | Not in English |
| 173 | Dib; M. | 2008 | Optimizing prophylactic treatment of migraine: Subtypes and patient matching |  | 0 | Not pertinent |
| 174 | D'Amico; D. et al. | 2008 | Does closure of a patent foramen ovale have a role in the treatment of migraine with aura? | 10.1007/s10072-008-0880-y | 0 | Not pertinent |
| 175 | Lucas; C. and Valade; D. | 2008 | Treatments of migraine |  | 0 | Not in English |
| 176 | Fumal; A. et al. | 2008 | Migraine management: Current trends and future prospects |  | 0 | Not in English |
| 177 | Lainez; J.M. et al. | 2007 | Recommendations guide for the treatment of migraine in the clinical practice | 10.1157/13101849 | 0 | Not in English |
| 178 | Fumal; A. and Schoenen; J. | 2008 | Current migraine management - Patient acceptability and future approaches |  | 0 | Not pertinent |
| 179 | Géraud; G. et al. | 2008 | SMILE: First observational prospective cohort study of migraine in primary care in France. Description of methods and study population | 10.1111/j.1468-2982.2007.01489.x | 0 | Not pertinent |
| 180 | Schürks; M. et al. | 2008 | Update on the prophylaxis of migraine | 10.1007/s11940-008-0003-3 | 0 | Not pertinent |
| 181 | Dodick; D.W. and Silberstein; S.D. | 2007 | Migraine prevention | 10.1136/jnnp.2007.134023 | 0 | Not pertinent |
| 182 | Hoffmann; J. and Reuter; U. | 2007 | Treatment of migraine | 10.1055/s-2007-985657 | 0 | Not in English |
| 183 | Diener; H.-C. and Limmroth; V. | 2005 | Migraine therapy | 10.1007/s00108-005-1459-9 | 0 | Not in English |
| 184 | Göbel; H. et al | 2006 | Prophylactic measures and acute treatment of migraine | 10.1007/s00482-006-0502-7 | 0 | Not in English |
| 185 | Benito-León; J. et al. | 2006 | Prophylactic treatment of migraine | 10.1157/13086327 | 0 | Not in English |
| 186 | Evers; S. et al | 2006 | EFNS guideline on the drug treatment of migraine - Report of an EFNS task force | 10.1111/j.1468-1331.2006.01411.x | 0 | Not pertinent/duplicate |
| 187 | Lucas; C. et al. | 2006 | Recognition and therapeutic management of migraine in 2004; in France: Results of FRAMIG 3; a French nationwide population-based survey | 10.1111/j.1526-4610.2006.00430.x | 0 | Not pertinent |
| 188 | Landy; S.H. and Lobo; B.L. | 2005 | Migraine treatment throughout the lifecycle | 10.1586/14737175.5.3.343 | 0 | Not pertinent |
| 189 | Strupp; M. and Brandt; T. | 2005 | Neurology - Current treatment options | 10.1055/s-2005-870858 | 0 | Not in English |
| 190 | Knuistingh Neven et al. | 2004 | Standard for headache of the Dutch College of General Practitioners |  | 0 | Not in English |
| 191 | Diener; H.-C. and Gendolla; A. | 2004 | Treatment and prophylaxis of an acute migraine attack |  | 0 | Not in English |
| 192 | Diener; H.-C. and Eikermann; A. | 2004 | Therapeutic Concepts in the Treatment of Headache |  | 0 | Not in English |
| 193 | Massiou; H. and Bousser; M.-G. | 2005 | Prophylactic drug treatment of migraine | 10.1016/s0035-3787(05)85116-1 | 0 | Not in English |
| 194 | Gobel; H et al. | 2002 | Prophylactic drug management of migraine | 10.1007/s00482-002-0146-1 | 0 | Not in English |
| 195 | Alves de Souza; J. | 2002 | Recommendations for prophylactic treatment of migraine | 10.1590/s0004-282x2002000100030 | 0 | Not in English |
| 196 | Elrington; G. | 2002 | Migraine: Diagnosis and management |  | 0 | Not pertinent |
| 197 | Dias Gherpelli; J.L. | 2002 | Treatment of headaches | 10.1590/s0021-75572002000700002 | 0 | Not in English |
| 198 | Massiou; H. | 2000 | Prophylactic treatments of migraine |  | 0 | Not in English |
| 199 | Diener; H.-C. and Kaube; H. and Limmroth; V. | 1998 | A practical guide to the management and prevention of migraine | 10.2165/00003495-199856050-00006 | 0 | Not pertinent |
| 200 | Dalessio; D.J. | 1990 | Aspirin Prophylaxis for Migraine | 10.1001/jama.1990.03450130093035 | 0 | Wrong publication type |
| 201 | Edmeads; J.G. | 1988 | Migraine |  | 0 | Not pertinent |
| 202 | Dalessio; D.J. | 1978 | Migraine; Platelets; and Headache Prophylaxis | 10.1001/jama.1978.03280280052030 | 0 | Wrong publication type |
| 203 | Mattle HP et al. | 2016 | Percutaneous closure of patent foramen ovale in migraine with aura; a randomized controlled trial. | 10.1093/eurheartj/ehw027 | 0 | Not pertinent |
| 204 | Evers S et al. | 2006 | EFNS guideline on the drug treatment of migraine - report of an EFNS task force. | 10.1111/j.1468-1331.2006.01411.x | 0 | Not pertinent |
| 205 | Edmeads J | 1991 | Migraine equivalents and complicated migraine. | 10.1016/s0025-7125(16)30433-3 | 0 | Not pertinent |
