# Supplementary material for: Do antithrombotic drugs have a role in migraine prevention? A systematic review
Source: Headache. 2025 Feb 24;65(4):709–27. doi: 10.1111/head.14917 (PMC11951400; doi:10.1111/head.14917)
Supplement: Supplementary file 2 — Table S2. [file HEAD-65-709-s002.docx]

**Supplemental table 2**. Risk of bias assessment of included randomized controlled trials performed according to the Cochrane Collaboration RoB-2 tool.

| **STUDY** | **DOMAIN** | **JUDGMENT** | **REASON** |
| --- | --- | --- | --- |
| ***Baldrati et al., 1982*** | 1: randomization process | 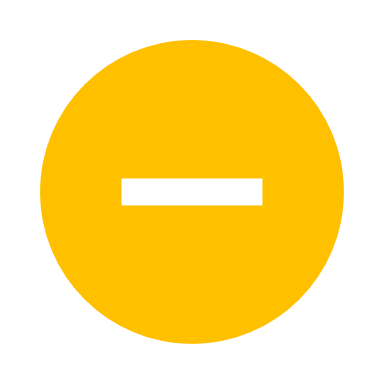 | Details on the sequence of randomization are not reported |
|  | 2: deviation from the intended deviation | 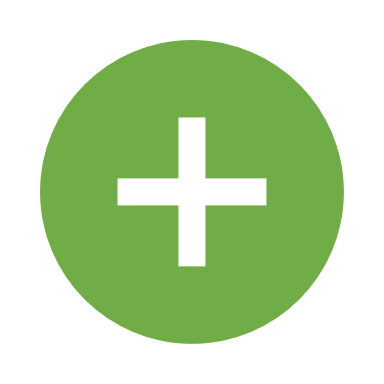 |  |
|  | 3: missing outcome data | 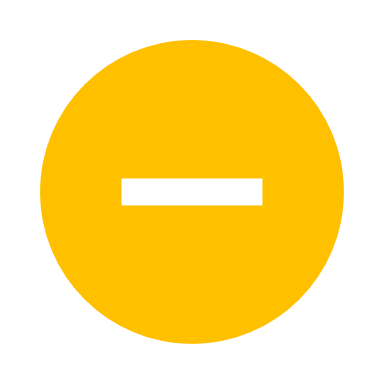 | Missing data from patient drop-outs; no sensitivity analysis or measure to correct bias |
|  | 4: measure of outcome | 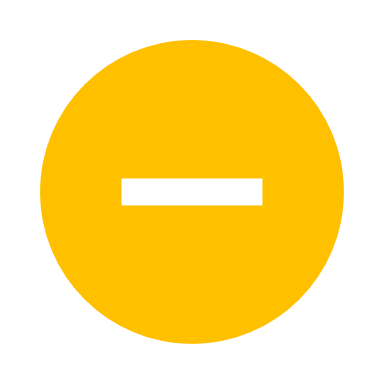 | Outcomes were reported using a migraine index that was not validated |
|  | 5: reported results | 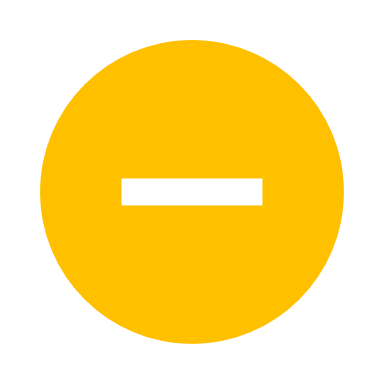 | Outcomes were presented only using a migraine index combining different outcomes a three months. |
| ***Bensenor et al., 2001*** | 1: randomization process | 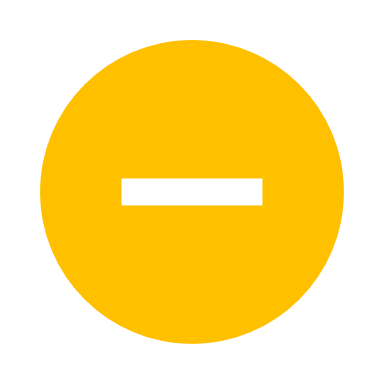 | The sequence of randomization and the method is not clear. |
|  | 2: deviation from the intended deviation | 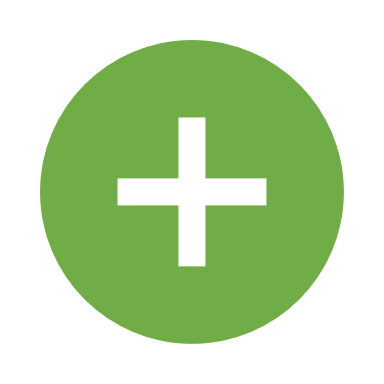 |  |
|  | 3: missing outcome data | 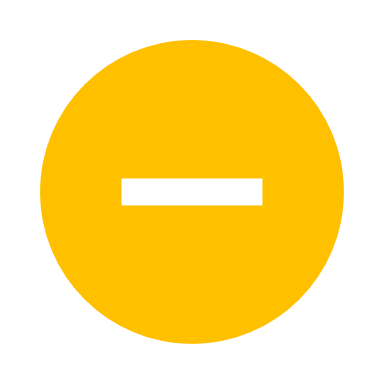 | Only a part of the population returned the questionnaires self-administered of migraine. |
|  | 4: measure of outcome | 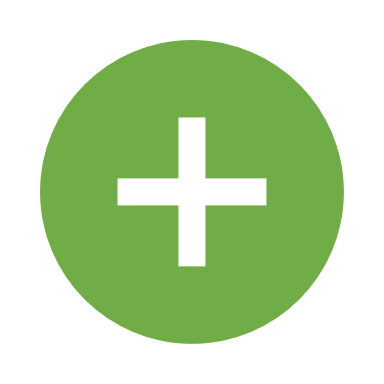 |  |
|  | 5: reported results | 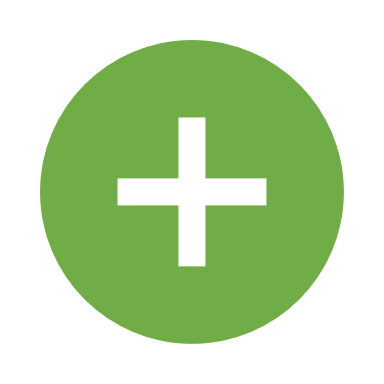 |  |
| ***Bousser et al., 1998*** | 1: randomization process | 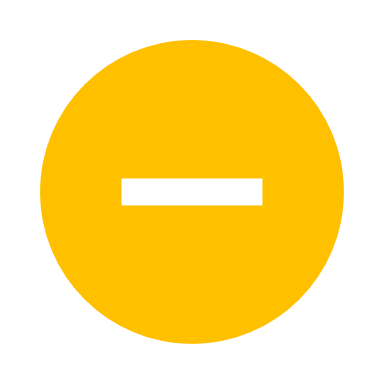 | Any details on the methods of randomization |
|  | 2: deviation from the intended deviation | 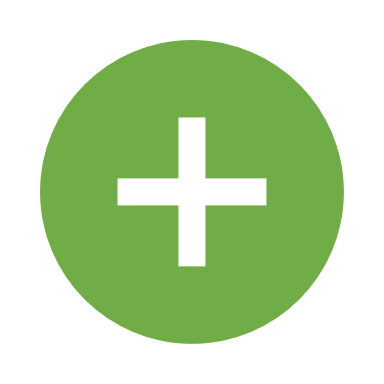 |  |
|  | 3: missing outcome data | 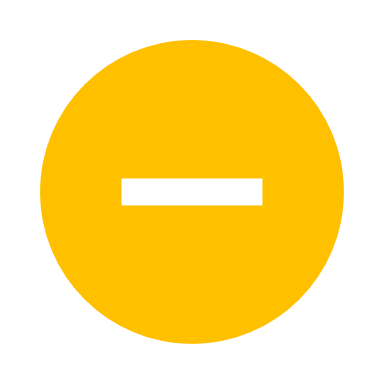 | Several missing data from the high rate of drop-out of patient during the follow-up. |
|  | 4: measure of outcome | 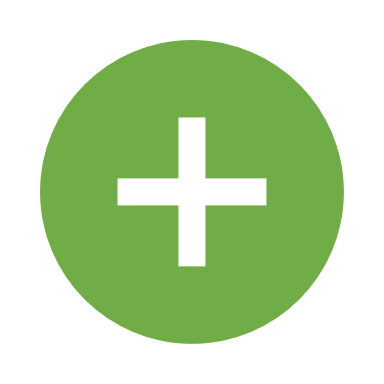 |  |
|  | 5: reported results | 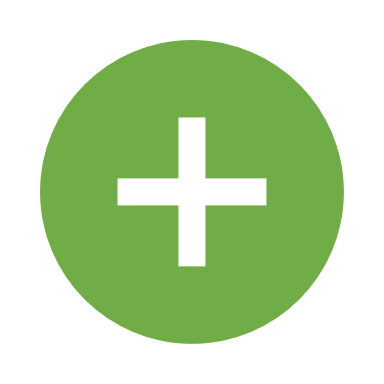 |  |
| ***Buring et al., 1990*** | 1: randomization process | 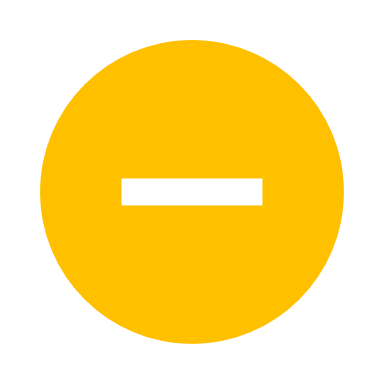 | Lack of data on the randomization process. |
|  | 2: deviation from the intended deviation | 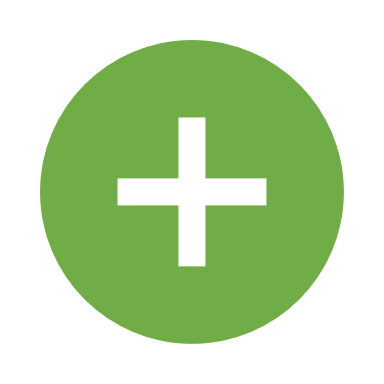 |  |
|  | 3: missing outcome data | 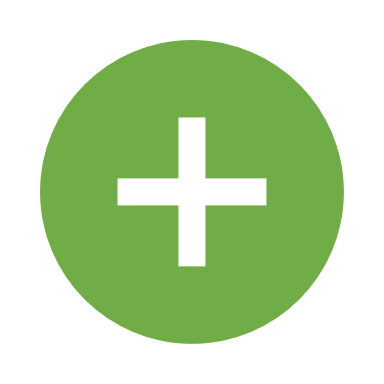 |  |
|  | 4: measure of outcome | 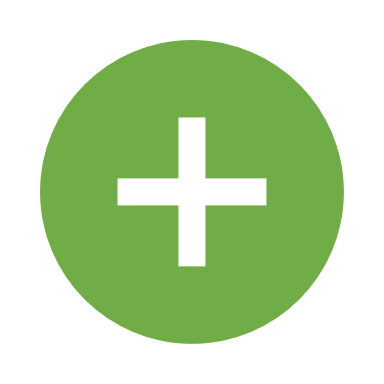 |  |
|  | 5: reported results | 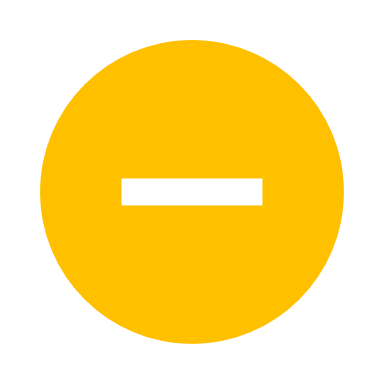 | *As a subgroup analysis of a trial, the analysis intention on migraine frequency was not specified.* |
| ***Chambers et al., 2014*** | All domain | 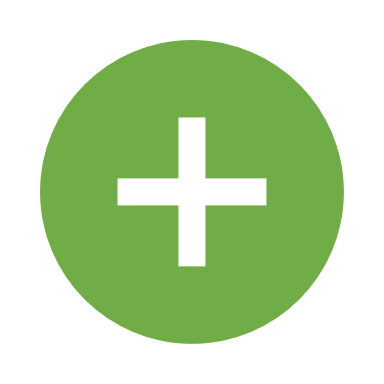 |  |
| ***Diener et al., 2001*** | 1: randomization process | 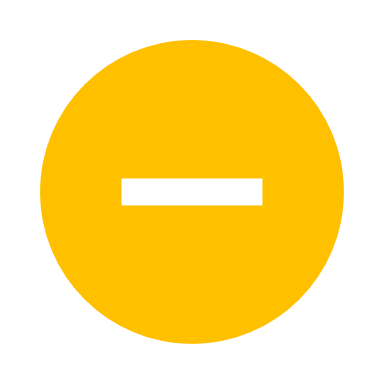 | Lack of data on the randomization process. |
|  | 2: deviation from the intended deviation | 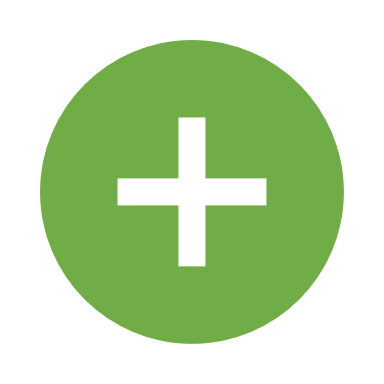 |  |
|  | 3: missing outcome data | 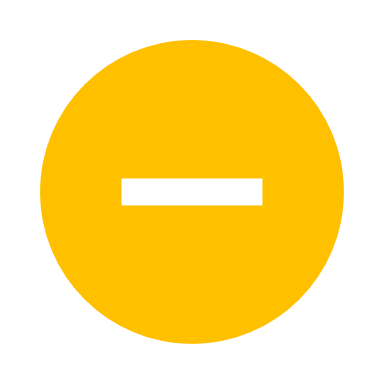 | Several patients excluded from the per protocol analysis due to incomplete data. |
|  | 4: measure of outcome | 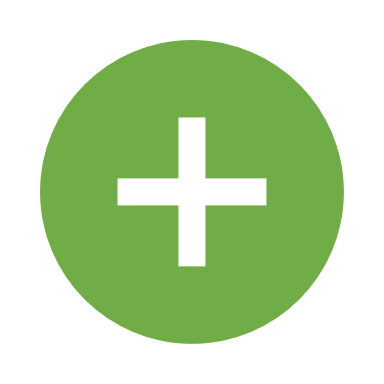 |  |
|  | 5: reported results | 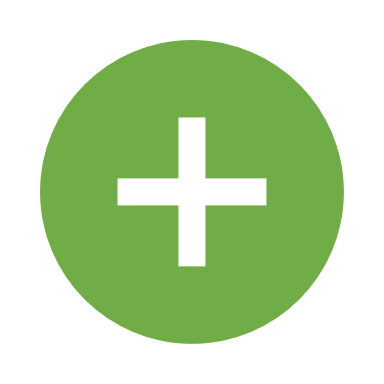 |  |
| ***Grotemeyer et al., 1990*** | 1: randomization process | 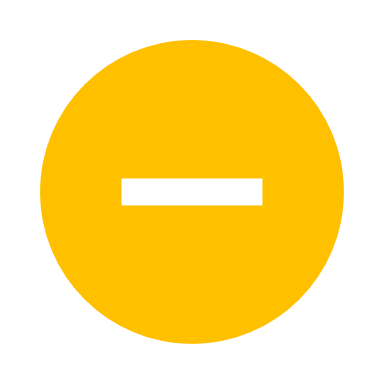 | Lack of data on the randomization process. |
|  | 2: deviation from the intended deviation | 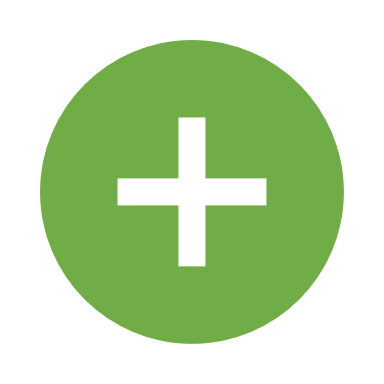 |  |
|  | 3: missing outcome data | 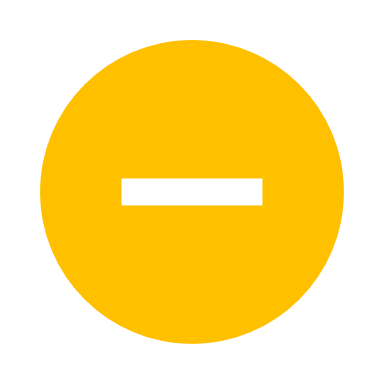 | Low number of patients, with high number of drop-out during the follow-up. |
|  | 4: measure of outcome | 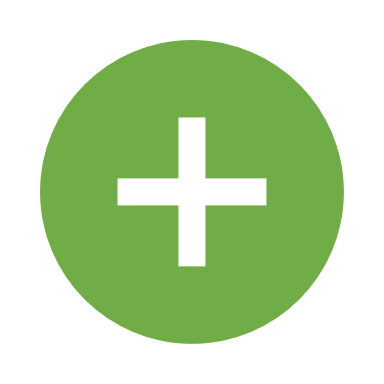 |  |
|  | 5: reported results | 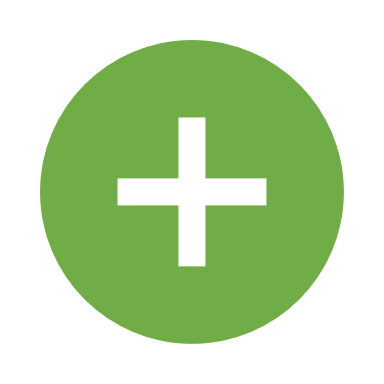 |  |
| ***Masel et al., 1980*** | 1: randomization process | 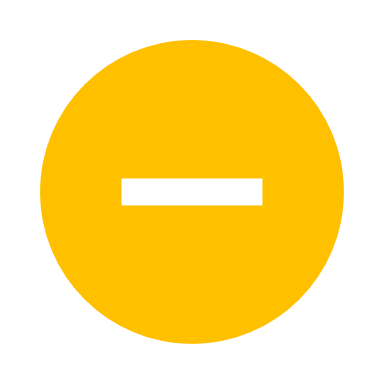 | Lack of data on the randomization process. |
|  | 2: deviation from the intended deviation | 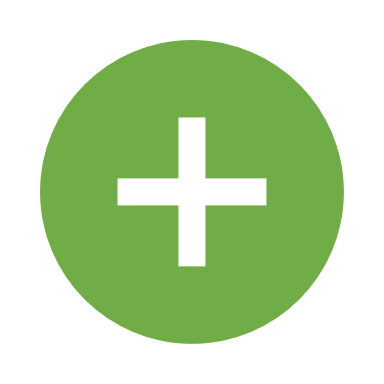 |  |
|  | 3: missing outcome data | 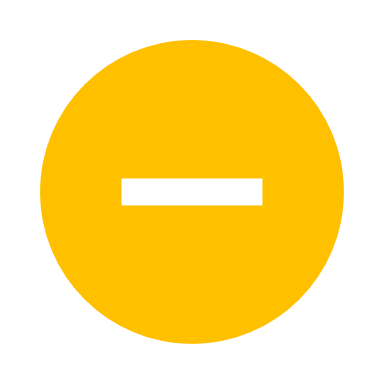 | Several data missing during the follow-up |
|  | 4: measure of outcome | 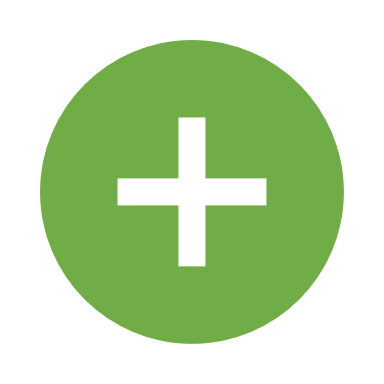 |  |
|  | 5: reported results | 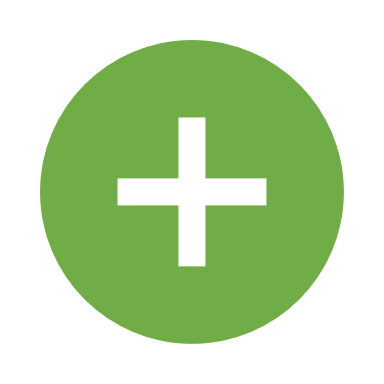 |  |
| ***O’Neill et al., 1978*** | 1: randomization process | 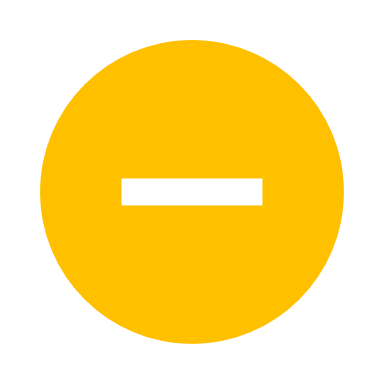 | No clear data on randomization process. |
|  | 2: deviation from the intended deviation | 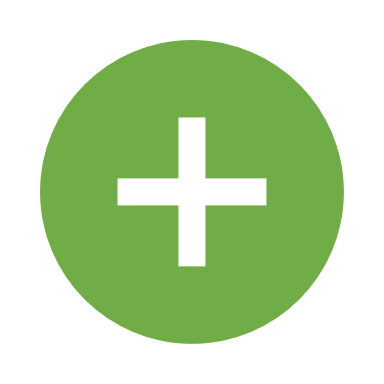 |  |
|  | 3: missing outcome data | 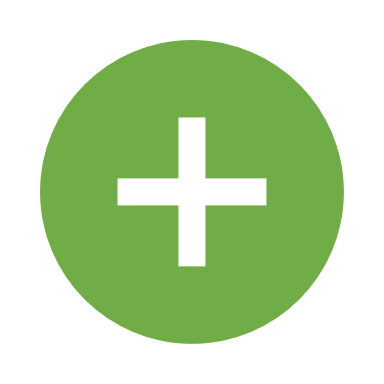 |  |
|  | 4: measure of outcome | 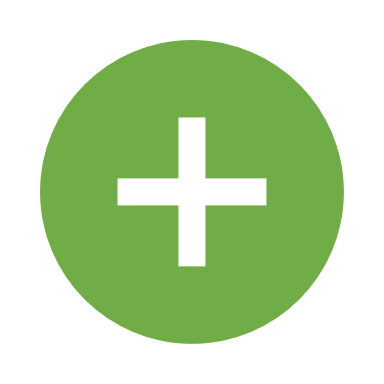 |  |
|  | 5: reported results | 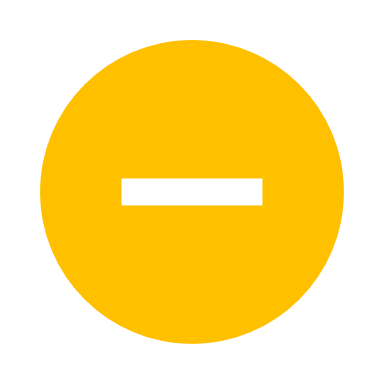 | No specific plan of analysis, a generic positive response was selected as main result. |
| ***Peto et al., 1988*** | 1: randomization process | 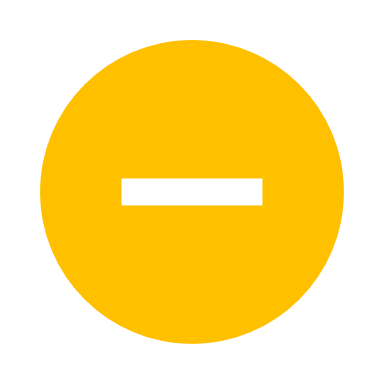 | Lack of data on the randomization process. |
|  | 2: deviation from the intended deviation | 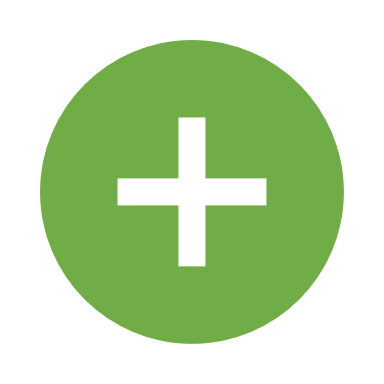 |  |
|  | 3: missing outcome data | 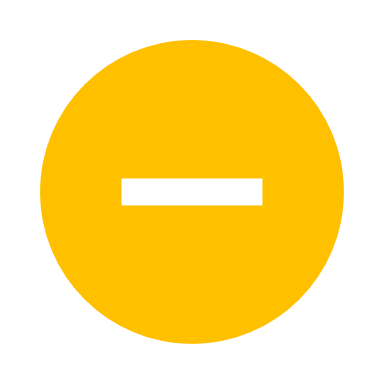 | The study was based on returned questionnaire every 6 months |
|  | 4: measure of outcome | 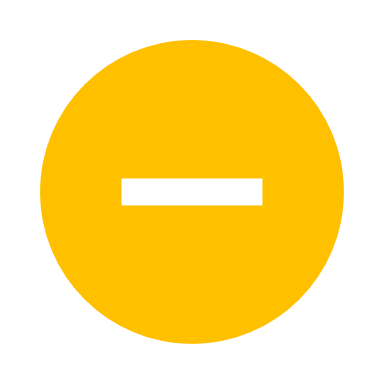 | The measure of outcome was not standardized |
|  | 5: reported results | 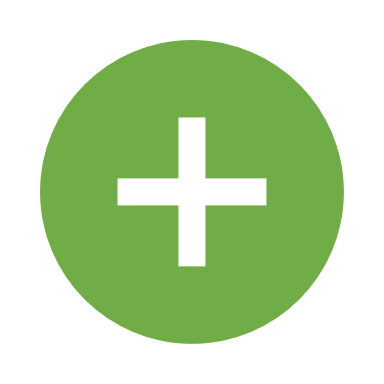 |  |
| ***Rodes-Cabau et al., 2015*** | 1: randomization process | 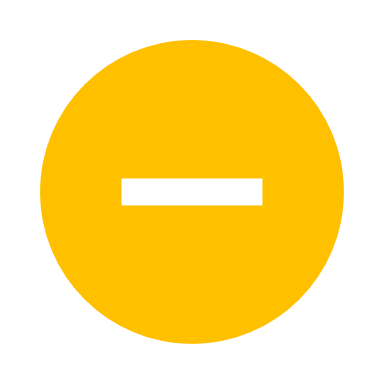 | Lack of data on the randomization process. |
|  | 2: deviation from the intended deviation | 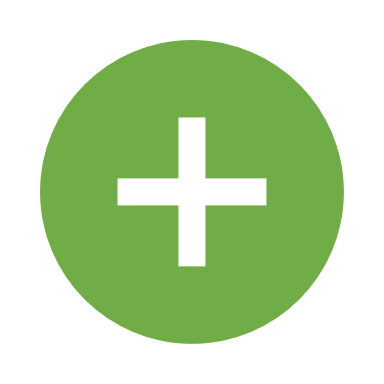 |  |
|  | 3: missing outcome data | 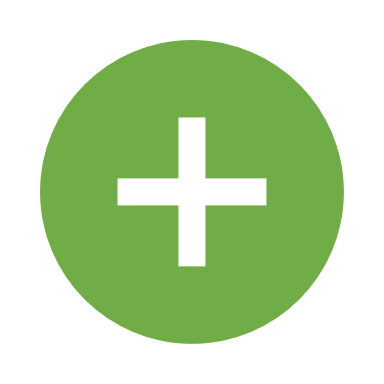 |  |
|  | 4: measure of outcome | 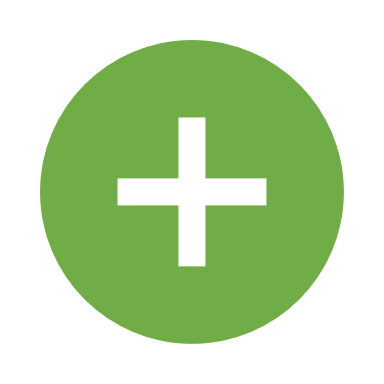 |  |
|  | 5: reported results | 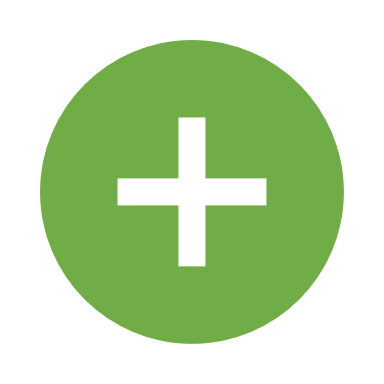 |  |
| ***Wammes – van der Heijde et al., 2005*** | 1: randomization process | 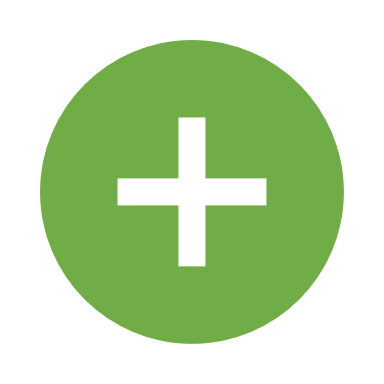 |  |
|  | 2: deviation from the intended deviation | 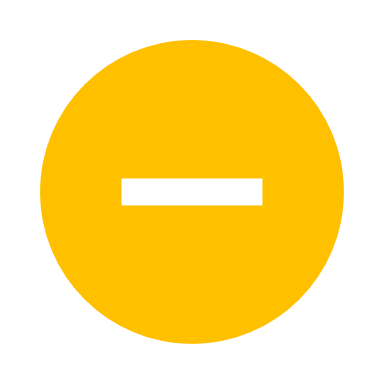 | Patients and investigators were aware of the type of interventions, several discontinuation for “private reasons” |
|  | 3: missing outcome data | 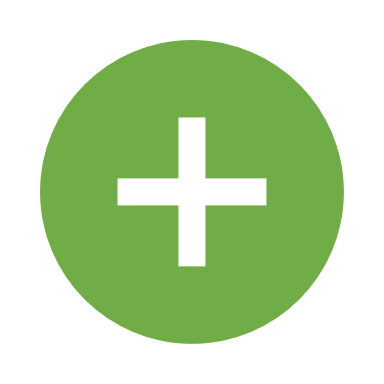 |  |
|  | 4: measure of outcome | 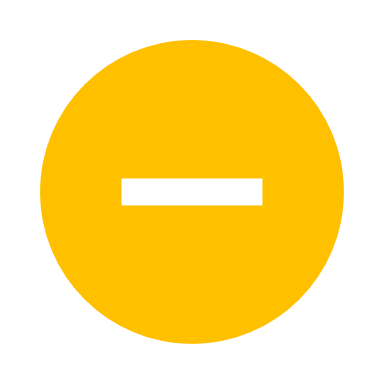 | The open design could influence the outcome assessment. |
|  | 5: reported results | 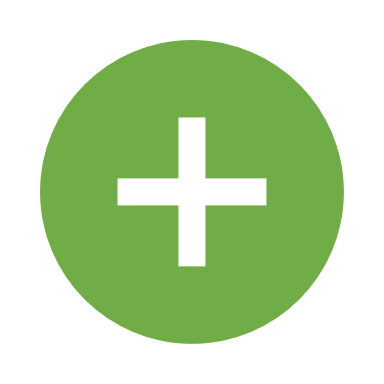 |  |

| 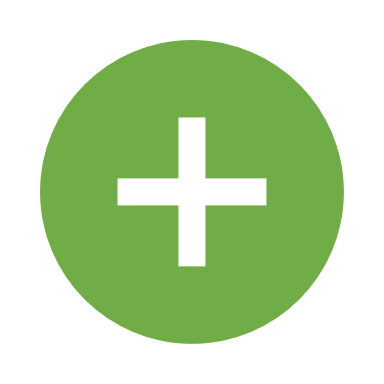 | Low risk |
| --- | --- |
| 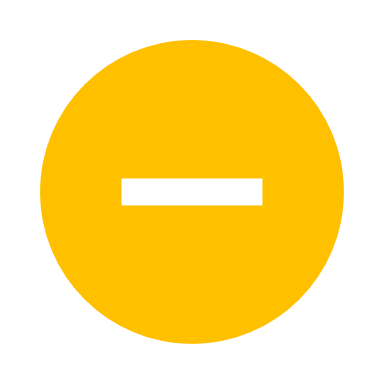 | Moderate risk/some concerns |
| 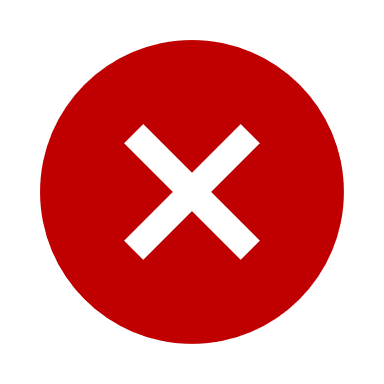 | High risk |

**Supplemental table 3**. Risk of bias assessment of observational studies included in the present systematic review, performed according to the Cochrane Collaboration ROBINS-I tool.

| **STUDY** | **DOMAIN** | **JUDGMENT** | **REASON** |
| --- | --- | --- | --- |
| ***Anoaica et al., 2014*** | 1: Bias due to confounding | 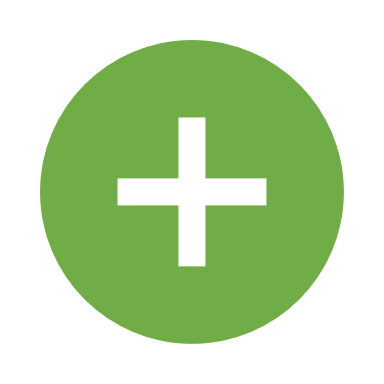 |  |
|  | 2: Bias in selection of participants | 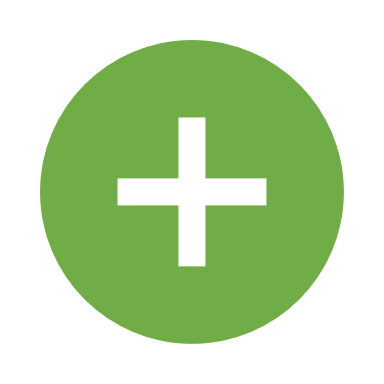 |  |
|  | 3: Bias in classification of interventions | 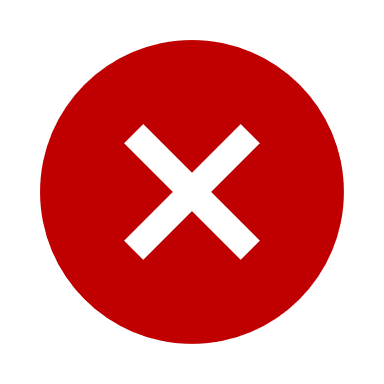 | Poor details on the classification of intervention in both groups (ASA vs other preventive drugs not specified) at variable dosages and duration of treatment. |
|  | 4: Bias due to deviations from intended interventions | 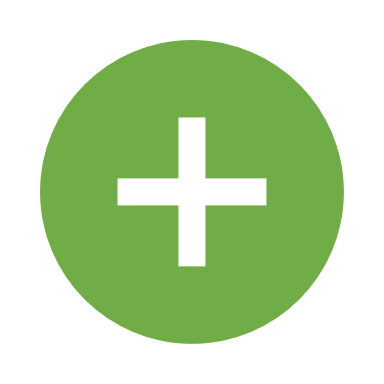 |  |
|  | 5: Bias due to missing data | 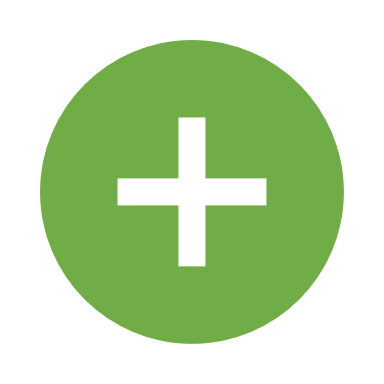 |  |
|  | 6: Bias in measurement of outcomes | 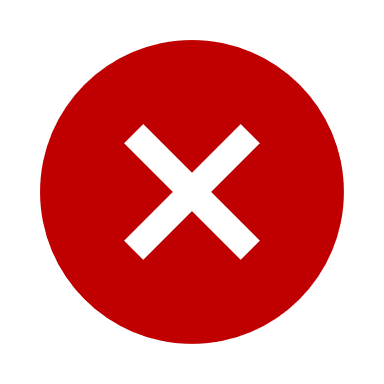 | Measurement of outcomes: methods of outcome assessment were not available, generic “positive results” without specifying the outcome. Not detailed information on the outcomes measure methods in the interventions groups. |
|  | 7: Bias in selection of the reported result | 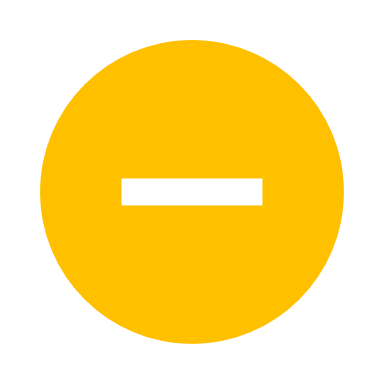 | Reported effect estimates likely to be selected, on the basis of the results, from multiple outcomes. |
| ***Guo et al., 2020*** | 1: Bias due to confounding | 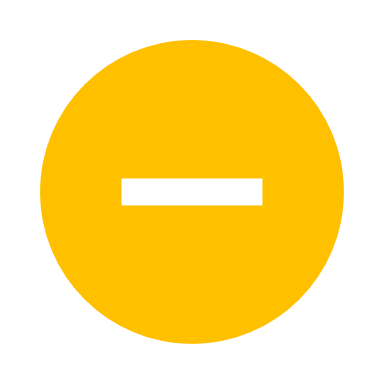 | Lack of control group led to baseline confounding bias, |
|  | 2: Bias in selection of participants | 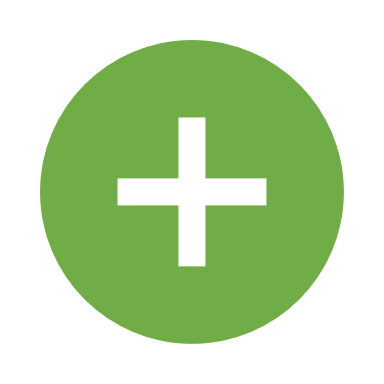 |  |
|  | 3: Bias in classification of interventions | 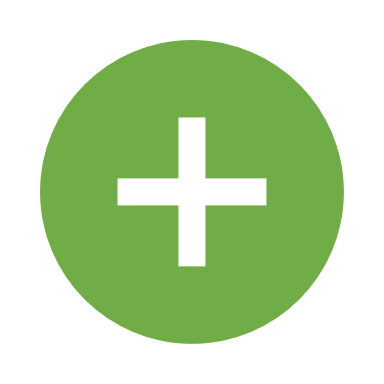 |  |
|  | 4: Bias due to deviations from intended interventions | 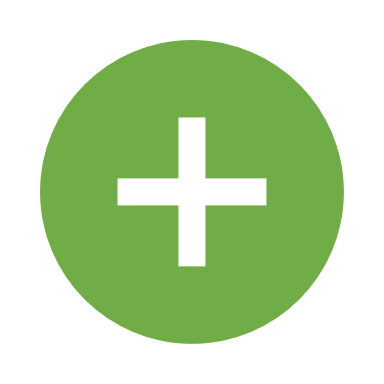 |  |
|  | 5: Bias due to missing data | 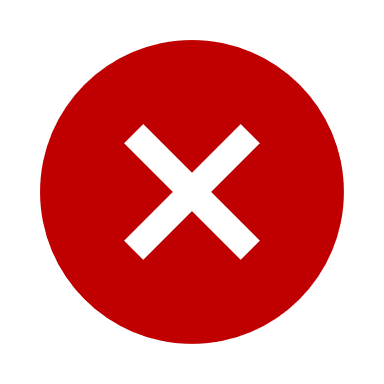 | High bias due to missing data: small sample size at baseline with missing data of follow-up data at 3 and 6 months. |
|  | 6: Bias in measurement of outcomes | 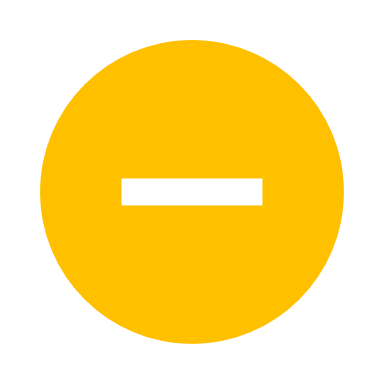 | Outcome measure could be influenced by patient and investigator knowledge. |
|  | 7: Bias in selection of the reported result | 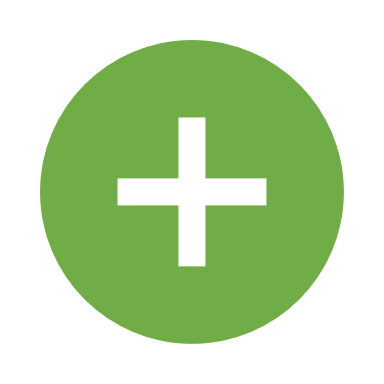 |  |
| ***Morales-Asín et al., 2000*** | 1: Bias due to confounding | 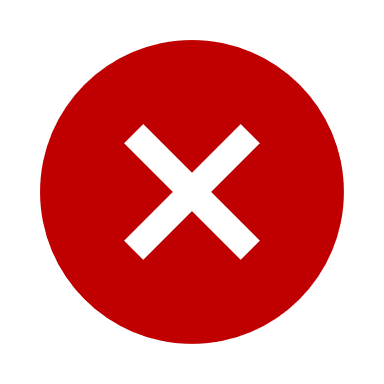 | High risk bias (confounding and selection): the study is a retrospective analysis based on questionnaire on headache in patient selected after starting the intervention for other reasons. |
|  | 2: Bias in selection of participants | 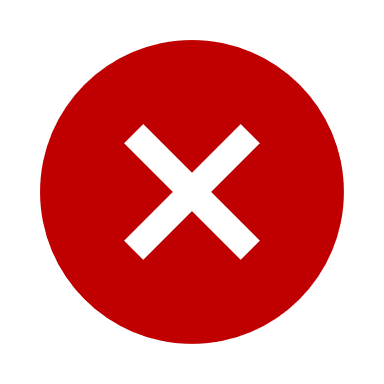 | Selection after starting interventions. |
|  | 3: Bias in classification of interventions | 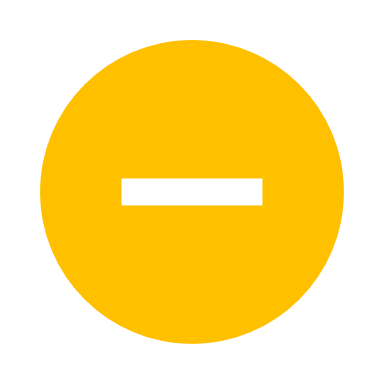 | No detailed data on the intervention (generic anticoagulation). |
|  | 4: Bias due to deviations from intended interventions | 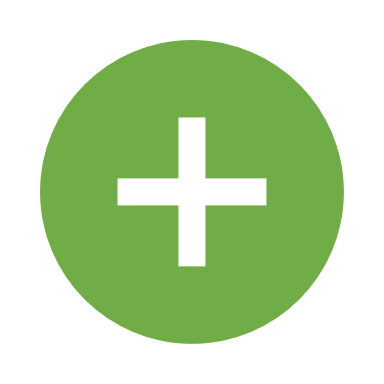 |  |
|  | 5: Bias due to missing data | 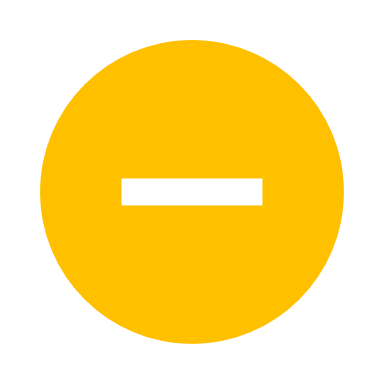 | Several missing data. |
|  | 6: Bias in measurement of outcomes | 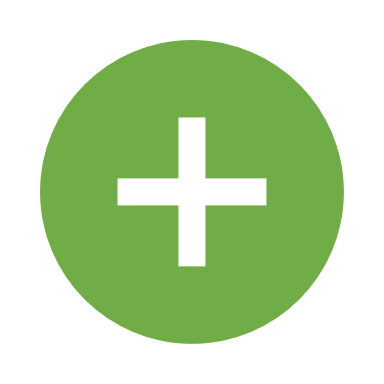 |  |
|  | 7: Bias in selection of the reported result | 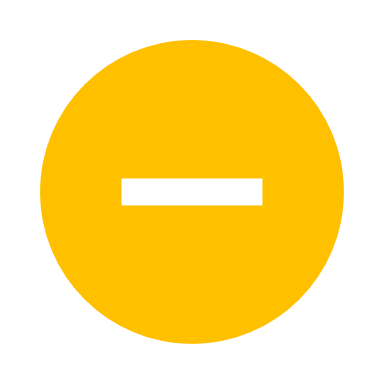 | Not clear the selection of reported results. |
| ***Rahimtoola et al., 2001*** | *Al domains:* | 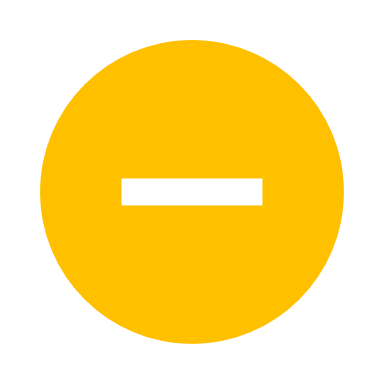 | Moderate risk of bias in all domains due to the nature of the study (a retrospective follow-up study based on a prescription database) with several confounding, selection. classification and outcome assessment biases. |
| ***Smith et al., 1984*** | 1: Bias due to confounding | 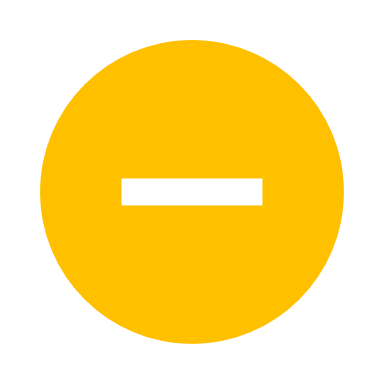 | No specific analysis to control potential baseline confounders (e.g. sex, age differences). |
|  | 2: Bias in selection of participants | 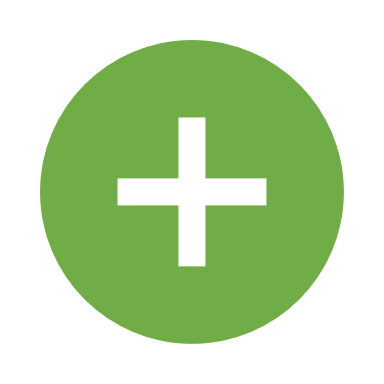 |  |
|  | 3: Bias in classification of interventions | 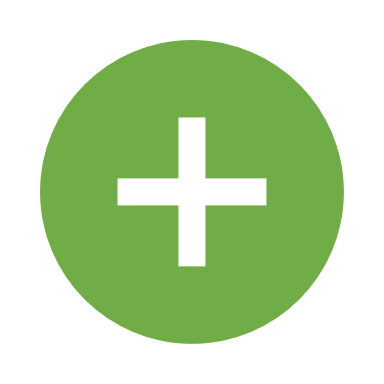 |  |
|  | 4: Bias due to deviations from intended interventions | 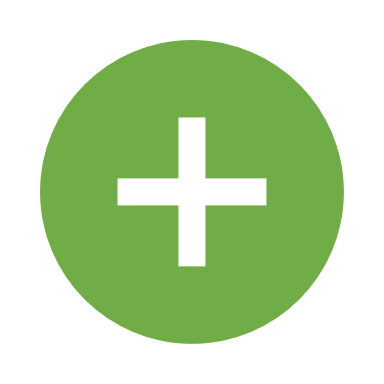 |  |
|  | 5: Bias due to missing data | 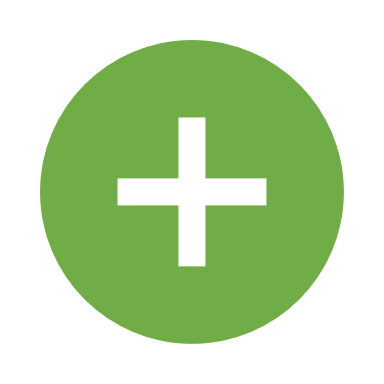 |  |
|  | 6: Bias in measurement of outcomes | 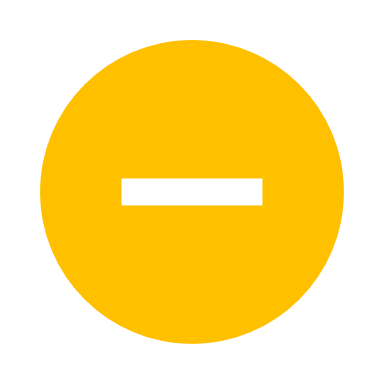 | The trial was open and the method and type of outcome assessment were not very clear. |
|  | 7: Bias in selection of the reported result | 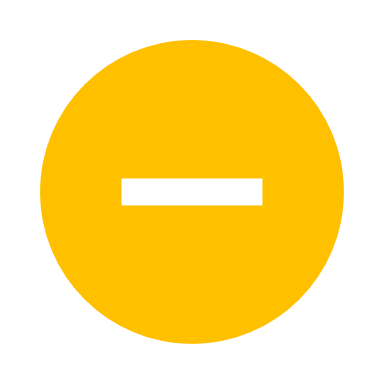 | No prespecified analysis of the results, the selection of outcome reported was not clear: they reported only the percentage of patient with levels of ”improvement” and “reduction of frequency” not described in the methods. |
| ***Spencer et al., 2014*** | All domain | 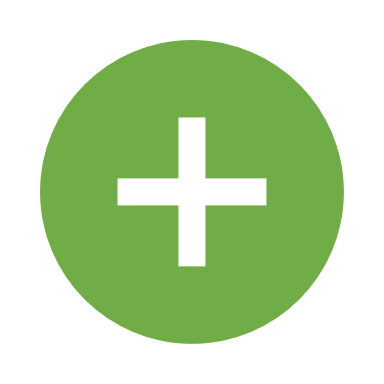 |  |
| ***Teber et al., 2007*** | 1: Bias due to confounding | 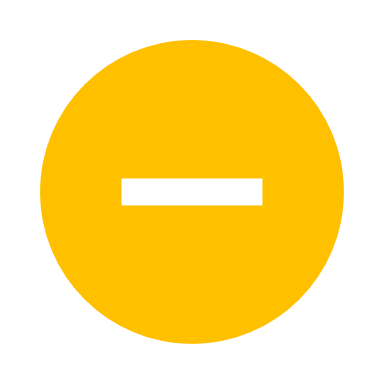 | Patient characteristics were very heterogeneous, no adjustments for confounding factors were reported. |
|  | 2: Bias in selection of participants | 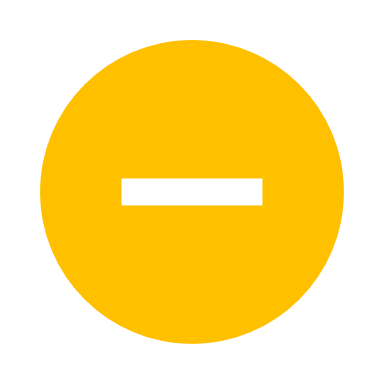 | Selection of patient and the start of the intervention was done after the start of the follow-up. |
|  | 3: Bias in classification of interventions | 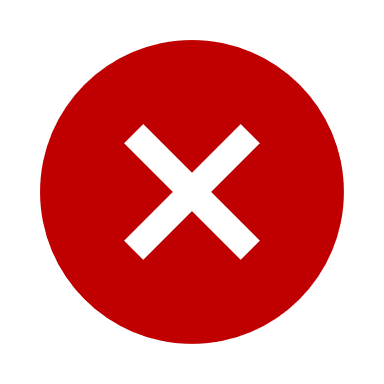 | Even if the intervention drug was well defined, the characteristics of the two intervention groups were not defined, one patient’s intervention was selected according to his vascular risk profile. |
|  | 4: Bias due to deviations from intended interventions | 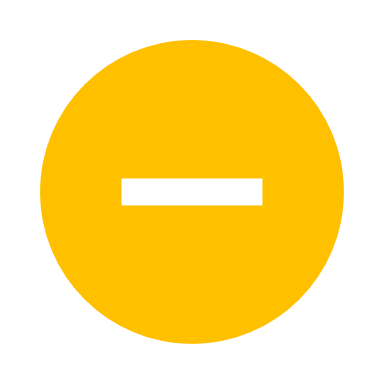 | Some deviations from the intended intervention were reported in both groups with early termination of the drug |
|  | 5: Bias due to missing data | 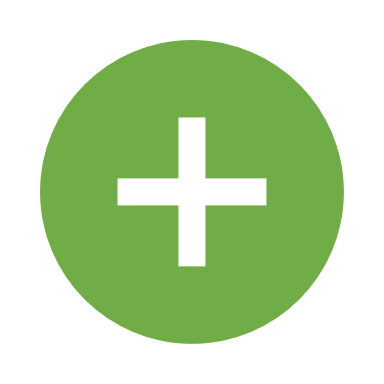 |  |
|  | 6: Bias in measurement of outcomes | 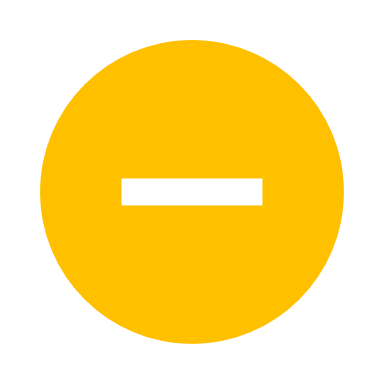 | Outcome measurement: both patient and evaluators were aware of the interventions with potential implication on the result |
|  | 7: Bias in selection of the reported result | 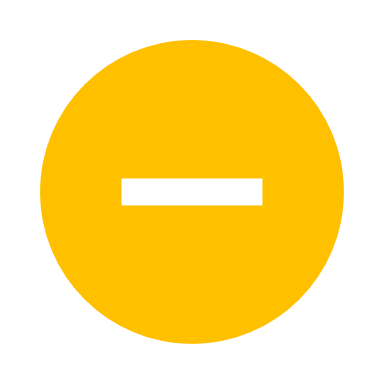 | The results relies only on differences in frequency, intensity and duration from the baseline. |
| ***Wammes – van der Heijde et al., 2004*** | 1: Bias due to confounding | 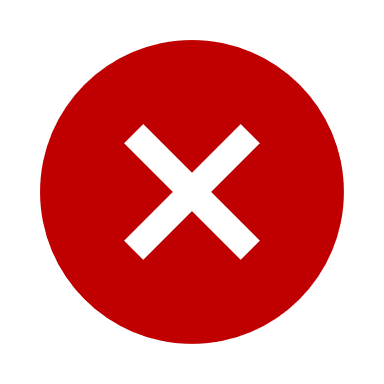 | Characteristics of intervention (INR range and dosage) and outcomes measure could be affected by the open design. |
|  | 2: Bias in selection of participants | 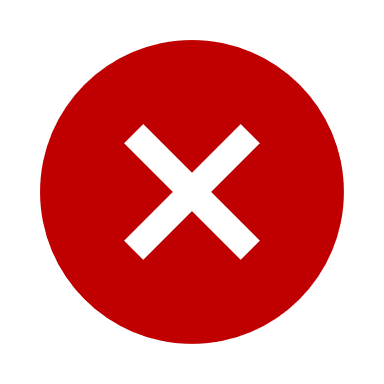 | The selection of participants is based on a previous intervention exposure and the related effect. |
|  | 3: Bias in classification of interventions | 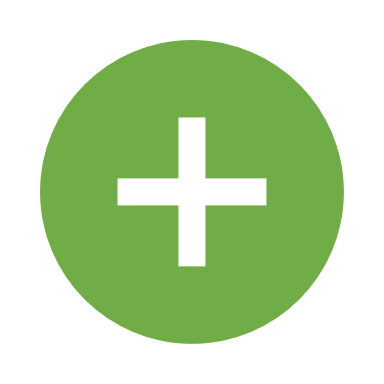 |  |
|  | 4: Bias due to deviations from intended interventions | 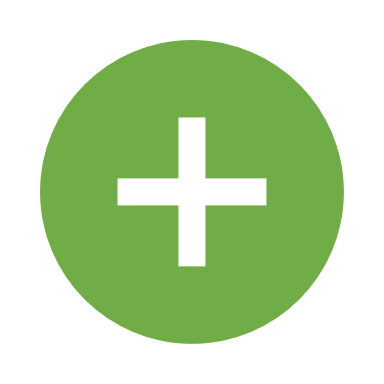 |  |
|  | 5: Bias due to missing data |  |  |
|  | 6: Bias in measurement of outcomes |  | Outcome reporting could be influenced by the open design. |
|  | 7: Bias in selection of the reported result |  | Different positive outcomes were selected for reporting results according to the thromboembolic risk profile. |

|  | Low |
| --- | --- |
|  | Moderate |
|  | Serious/critical |
